# The Apoplastic Secretome of *Trichoderma virens* During Interaction With Maize Roots Shows an Inhibition of Plant Defence and Scavenging Oxidative Stress Secreted Proteins

**DOI:** 10.3389/fpls.2018.00409

**Published:** 2018-04-05

**Authors:** Guillermo Nogueira-Lopez, David R. Greenwood, Martin Middleditch, Christopher Winefield, Carla Eaton, Johanna M. Steyaert, Artemio Mendoza-Mendoza

**Affiliations:** ^1^Bio-Protection Research Centre, Lincoln University, Lincoln, New Zealand; ^2^School of Biological Sciences, The University of Auckland, Auckland, New Zealand; ^3^Department of Wine, Food and Molecular Biosciences, Lincoln University, Lincoln, New Zealand; ^4^Bio-Protection Research Centre, New Zealand and Institute of Fundamental Sciences, Massey University, Wellington, New Zealand; ^5^Lincoln Agritech Ltd., Lincoln, New Zealand

**Keywords:** peroxidases, apoplast, reactive oxygen species (ROS), secretome, *Trichoderma*, roots, endophyte

## Abstract

In Nature, almost every plant is colonized by fungi. *Trichoderma virens* is a biocontrol fungus which has the capacity to behave as an opportunistic plant endophyte. Even though many plants are colonized by this symbiont, the exact mechanisms by which *Trichoderma* masks its entrance into its plant host remain unknown, but likely involve the secretion of different families of proteins into the apoplast that may play crucial roles in the suppression of plant immune responses. In this study, we investigated *T. virens* colonization of maize roots under hydroponic conditions, evidencing inter- and intracellular colonization by the fungus and modifications in root morphology and coloration. Moreover, we show that upon host penetration, *T. virens* secretes into the apoplast an arsenal of proteins to facilitate inter- and intracellular colonization of maize root tissues. Using a gel-free shotgun proteomics approach, 95 and 43 secretory proteins were identified from maize and *T. virens*, respectively. A reduction in the maize secretome (36%) was induced by *T. virens*, including two major groups, glycosyl hydrolases and peroxidases. Furthermore, *T. virens* secreted proteins were mainly involved in cell wall hydrolysis, scavenging of reactive oxygen species and secondary metabolism, as well as putative effector-like proteins. Levels of peroxidase activity were reduced in the inoculated roots, suggesting a strategy used by *T. virens* to manipulate host immune responses. The results provide an insight into the crosstalk in the apoplast which is essential to maintain the *T. virens*-plant interaction.

## Introduction

*Trichoderma* spp. are cosmopolitan soil fungi with the capacity to establish symbiotic relationships within the roots of most plant species (Harman et al., 2004). *Trichoderma* spp. promote plant growth, increase nutrient availability, improve crop production, and increase sensitivity to respond successfully to later pathogen invasion termed as induced systemic resistance (ISR) (Shoresh and Harman, 2008; Vinale et al., 2008; Shoresh et al., 2010). Despite of the direct benefits obtained from this mutualistic interaction, plants react to endophyte colonization via a basal immune response activation, in which, plants have evolved different strategies to recognize conserved microbial features referred to as microbe-associated molecular patterns (MAMPs) (Lorito et al., 2010; Zamioudis and Pieterse, 2012; Schmoll et al., 2016; Mendoza-Mendoza et al., 2018). Diverse MAMPs synthetized by *Trichoderma* have been identified (Hermosa et al., 2013), including the cerato-platanin protein Sm1 (Djonović et al., 2006) and the ethylene-inducing xylanase (EIX) (Ron and Avni, 2004). The proteinaceous elicitor Sm1 is induced during *Trichoderma virens-*plant interaction, which promotes the expression of pathogenesis-related genes (Djonović et al., 2007). The EIX has a dual role during plant colonization, involving both lytic enzyme activity and induction of systemic resistance in specific cultivars of tobacco and tomato (Rotblat et al., 2002; Ron and Avni, 2004).

Endophytic *Trichoderma* penetrates the first or second layers of plant root systems, first colonizing the root epidermis and then into the cortex area, without reaching the vascular system (Chacón et al., 2007). The initial steps of root colonization by *Trichoderma* starts with the attachment on the root surface followed by the formation of appressoria-like structures that may help for the penetration into the internal tissues (Yedidia et al., 1999, 2000; Viterbo and Chet, 2006). After recognition by *Trichoderma* MAMPs, the plant responds by depositing callose in the neighborhood cells allowing only superficial cell-colonization. Microscopic observations of early colonization of tomato roots by *T. harzianum* showed the capacity of the fungus to colonize inter- and intracellular spaces without disrupting cell integrity (Chacón et al., 2007). However, *T. asperellum* (formally called *T. harzianum*) induces morphological and physiological changes in cucumber plant roots, which includes necrosis of the penetration peg, high chitinase activity and formation of fluorescent products in intercellular spaces of the colonized roots (Yedidia et al., 1999). Additionally, enhanced protection against reactive oxygen species (ROS), and repression of the ethylene synthesis pathway is proposed to enable root colonization by *Trichoderma* as has been shown in other endophytes (Shoresh et al., 2010). Recently, was observed that the cerato-platanin elicitor Sm2 from *T. virens* is required for root colonization (Crutcher et al., 2015), although the mode of action is currently unknown.

Plant symbionts and pathogens have developed specific strategies to promote colonization by evading the first layer of plant defense called MAMP-triggered immunity (MTI). Plant microbes secrete molecules including effector proteins into the apoplast where they interact with their molecular targets or are translocated into the plant cell cytoplasm blocking downstream signals, thereby suppressing MTI. Examples of conventional secreted effectors, including the *Cladosporium fulvum* LysM effector Ecp6 which prevents elicitation of host defense by sequestering chitin oligosaccharides of the fungus (de Jonge et al., 2010), and the toxin-like ToxB which is secreted into the apoplast by the necrotrophic fungus *Pyrenophora tritici-repentis* and is necessary for complete disease development in wheat (Figueroa et al., 2015). Furthermore, unconventionally secreted effector proteins also play important roles in the manipulation of plant processes. The protein chorismate mutase Cmu1, secreted by *Ustilago maydis*, manipulates the metabolome of neighboring cells to favor pathogen infection (Djamei et al., 2011).

Recent studies have focused on the secretome of *T. virens* to the presence of maize roots (Lamdan et al., 2015). However, proteins from the apoplastic region, where *T. virens* closely interacts with host cells, were not considered in this study. Plant-associated microbes and their host plants continuously secrete an arsenal of proteins into the apoplast using a conventional secretion system, which involves the Golgi-endoplasmic reticulum pathway an approach for those proteins that carry an N-terminal signal peptide. Apoplastic proteins (APs) are also delivered into the apoplast by leaderless secretory pathways (LSPs) that constitutes, on average, 50% of the plant and fungal secretomes (Agrawal et al., 2010; Ding et al., 2012; Girard et al., 2013; Delaunois et al., 2014). APs secreted by both players have major roles in the maintenance of plant cell wall structure, stress responses, primary and secondary metabolism, defense and signaling (Alexandersson et al., 2013; Kim et al., 2013).

Proteomic tools such as mass spectrometry (MS) enables identification of key proteins of the secretome during complex physiological cell processes such as microbe-host interactions (Schmidt and Volker, 2011; Delaunois et al., 2014; Gupta et al., 2015). One clear example is the study of the apoplastic secretome of the phytopathogenic fungus *Magnaporthe oryzae* during interaction with rice plants, where Kim et al. (2013) identified more than 200 proteins secreted into the apoplast including putative effector proteins. Here we present the morphological changes occurring in the maize roots by their interaction with *T. virens* Gv29.8 in a sterile system, then we identified the fungal colonization to the plant root tissue and finally we analyzed the apoplastic secretome during the interaction between *T. virens* and maize roots by using two different approaches: (1) gel-based, sodium dodecyl sulfate polyacrylamide gel electrophoresis (SDS-PAGE) couple with LC-MS/MS which allows separation and identification of a large number of proteins (Gel-LC-MS/MS), and (2) gel-free shotgun proteomics, which is more powerful technology for large-scale separation and identification of complex mixtures of proteins (González-Fernández et al., 2010; Porteus et al., 2011; Jayaraman et al., 2012). The results will provide a better understanding of how endophytic *T. virens* modulates host plant defensive processes and how plant responds to the presence of the fungus.

## Materials and methods

### Maize germination

Maize seeds from hybrid line 34H31 (Pioneer® Brand Products, Gisborne, New Zealand) were surface sterilized by soaking in 2% (w/v) sodium hypochlorite (NaOCl) (active ingredient) for 7 min, followed by 70% ethanol for 7 min, then washed three times with sterile nanopure water. Seeds were germinated on sterile seed germination papers (30 × 45 cm; Anchor Paper Company, MN, USA) previously soaked in sterile Hoagland's No.2 basal salt solution (Sigma-Aldrich, MO, USA) (Hoagland and Arnon, 1950), for 60 h in a humidity controlled plant growth chamber at 25°C under a 16 h light/8 h dark cycle and a relative humidity of 80%.

### Inoculum preparation

*T. virens* Gv 29.8 conidia was propagated on potato-dextrose agar (PDA) (Difco, Fisher Scientific, NH, USA) at 25°C under a cycle of 12 h light and 12 h dark for 7 d to induce conidiation. Conidia were collected using sterile nanopure water and filtered through a double layer of sterile of Miracloth (Millipore Merck, MA, USA).

### Colonization of maize roots by *Trichoderma virens*

Sterilized maize seeds were surface inoculated with 1 × 10^6^ conidia. After germination, seedlings were grown under hydroponic conditions without aeration in 50 mL centrifuge tubes containing 45 mL of sterile Hoagland's solution with a piece of sterile cotton to support the seedling. Seedlings were incubated for an additional 60 h as described by Lawry (2016). For sampling, fresh root tissues were washed gently with sterile nanopure water, and then 2 cm sections nearest to the seed were cut from the primary root where *T. virens* primarily colonize (Lawry, 2016). Un-inoculated roots samples were taken as control.

### Confocal visualization of maize roots colonization by *Trichoderma virens*

*T. virens* root colonization of maize seeds was examined using confocal microscopy (Fluoview FV10i, Olympus, Tokyo, Japan). For this analysis, transverse free-hand sections of maize roots were prepared. After 5 d post inoculation (d.p.i) maize roots were collected and either washed in phosphate-buffered saline (PBS) pH 7.4 or fixed in fresh ethanol: acetic acid (3:1, v/v) solution. Two staining methods were used: for fresh tissues a wheat germ agglutinin (WGA)-Alexa Fluor™ 488 (Thermo Fisher Scientific, MA, USA) /FM4-64 dye (Thermo Fisher Scientific, USA) mixture was used, while fixed tissues were stained with WGA-Alexa Fluor™ 488/Propidium iodide (PI) (Sigma-Aldrich, USA) mixture. Fungal material was stained with WGA-Alexa Fluor™ 488 (Mochizuki et al., 2011). Plant cell walls were stained with PI, while the plasma membranes were stained with FM4-64 (Bolte et al., 2004).

For fixed tissues, roots were treated with 10% KOH for 4 h at 95°C and then transferred to PBS pH 7.4 for 1 h. Samples were infiltrated with the staining solution (20 μg/mL PI; 10 μg/mL WGA-Alexa Fluor™ 488, 0.02% Tween 20 made up in 1X PBS) for 15 min twice. Samples were distained in PBS-tween (0.02%) and stored in the dark at 4°C. For fresh tissues, samples were washed in PBS solution and infiltrated with the same staining solution mentioned above, except that PI was substituted for 5 mM FM4-64.

### Isolation of total protein from maize primary root

For total protein extraction from maize roots the methodology described by Wu et al. (2014) was used with modifications as follows. Fresh root tissue (0.25 g) was ground into a fine powder in liquid nitrogen, then homogenized in 2.5 mL of ice cold Tris/ethylenediaminetetraacetic acid (EDTA) extraction buffer, containing 1 mM EDTA, 10 mM Tris-HCL pH 8, 2% w/v polyvinylpolypyrrolidone (PVPP) and with 0.3% (v/v) Pefabloc (Sigma-Aldrich, USA). Samples were centrifuged at 5,000 × g for 30 min at 4°C, then supernatant proteins were precipitated with 10 mL of cold trichloroacetic acid (TCA)/acetone (−20°C). After centrifugation at 3,000 × g for 10 min, the pellet was washed three times with ice cold acetone containing 0.007% w/v dithiothreitol (DTT). Protein pellets were dried to evaporate any remaining acetone and stored at −80°C.

### Isolation of apoplastic proteins

For isolation of apoplastic fluid (AF), the primary root on the proximal side nearest to the seed (2 cm section) was cut from one side and collected using preferably the infiltration-centrifugation methodology (see Supplementary Methods). Individual primary roots from 20 plants were combined for each replicate. Three replicates were used for each condition: (a) inoculated and (b) un-inoculated plants. Roots were sampled 5 d.p.i.

Primary root sections (2 cm) were placed immediately in 100 mM sodium phosphate buffer (SPB) pH 6.5 (Witzel et al., 2011) supplemented with 0.3% (v/v) Pefabloc and 10 mM EDTA. The chilled samples were vacuum infiltrated by reducing the pressure at −45 kPa for 15 min using a diaphragm vacuum pump (Rocker 400, Rocker Scientific, Taipei, Taiwan), followed by slow return to atmospheric pressure to avoid cell damage (Dragišić Maksimović et al., 2008). Roots were then placed in a 5 mL syringe without the plunger and placed inside a 15 mL centrifuge tube then centrifuged at 2,000 × g for 15 min at 4°C (Model 5810R, Eppendorf, Hamburg, Germany). The harvested AF was filtered through cellulose acetate membrane filters (0.2 μm porosity; Axiva Sichem Biotech, Delhi, India) and stored at −80°C.

APs were concentrated using the TCA-sodium deoxycholate (Na-DOC)/acetone method described by Bensadoun and Weinstein (1976) with modifications. Briefly, for every volume of AF solution, 0.1 vol. of 2% of Na-DOC and 100% TCA were added and the samples were kept at RT for 1 h. Samples were then centrifuged at 14,000 × g for 10 min at 4°C, the supernatants removed and the pellet dried. The pellet was then washed in 200 μL of ice-cold acetone, placed on ice for 15 min, then centrifuged at 14,000 × g for 10 min at 4°C. The pellet was then air dried before being re-suspended in 10 μL in PBS buffer pH 7.0.

For gel-free shotgun proteomics, modifications were carried out to improve the method described above. Root sections were placed immediately in 50 mM potassium phosphate buffer (PPB) pH 5.5 supplemented with 0.3% (v/v) Pefabloc and 10 mM EDTA. Roots were then vacuum infiltrated with the PPB solution. The root samples were centrifuged at 2,000 × g for 15 min at 4°C (Dragišić Maksimović et al., 2008) and harvested AF was immediately snap frozen in liquid nitrogen. APs samples from un-inoculated and inoculated roots were concentrated by freeze drying (Thermo Savant Micro Modulyo-115, Thermo Fisher Scientific, USA). Samples were rehydrated in 30 μL of 50 mM ammonium acetate buffer pH 5.5 for quantification and then stored at −80°C.

### Malate dehydrogenase activity

Malate dehydrogenase (MDH) activity was performed as described by Husted and Schjoerring (1995) with modifications. MDH activity was assayed to determine cytoplasmic contamination in AF. A total of 5 μg of protein extract (total or apoplastic) were added into 3 mL reaction mixtures containing 0.094 mM β-NADH (Sigma-Aldrich, USA), 0.17 mM oxaloacetic acid (Sigma-Aldrich, USA), and 0.1 M phosphate buffer, pH 7.5. Oxidation of NADH was measured at 340 nm using an UV-Vis spectrophotometer (Genesys 10S, Thermo Fisher Scientific, USA), monitoring for 5 min at 25°C. A non-enzyme reaction mix was used as a blank. Enzymatic activity in AF was expressed as a percentage of the total root protein extract activity.

### Identification of apoplastic proteins by Gel-LC-MS/MS

APs were separated on a 4–12% pre-cast NuPAGE bis-tris gel (Novex, Life Technologies, CA, USA) in 1X MOPS SDS running buffer (2.5 mM MOPS, 2.5 mM Tris, 0.005% SDS, 0.05 mM EDTA, pH 7.7). Prior to loading, samples were mixed with 2 μL 6X Tris-Glycine SDS sample buffer (0.378 M Tris-HCl pH 6.8, 0.6 M DTT, 12% SDS, 60% Glycerol, 0.06% Bromophenol Blue). Gels were stained with Coomassie blue solution (10% acetic acid, 50% methanol, 0.25% Coomassie blue R-250). Molecular weights were determined using the SDS-PAGE PageRuler Plus prestained protein ladder (Fermentas, MA, USA).

For identification, appropriate protein zones were excised and subjected to trypsin digestion. Briefly, excised gel plugs were washed three times with 200 μL of 1:1 acetonitrile: 50 mM ammonium bicarbonate (ABC) solution pH 8.3, and dried in a vacuum centrifuge. The plugs were then incubated with 10 mM DTT in 50 mM ABC solution pH 8.3 for 1 h at 56°C. Each plug was diced into small cubes with dimensions of 0.5–1.0 mm. For distaining, 200 μL of 50% acetonitrile (ACN)/ 50 mM ABC was added and the tubes were vortexed for 30 s. Plugs were then incubated at 45°C for 15 min in a Discoverer II System microwave (CEM Corporation, NC, USA). For gel dehydration and reduction, 200 μL of 100% ACN was added and the tubes vortexed for 30 s and placed in a heating block (Thermomixer, Eppendorf, USA) at 56°C with the lids open to allow ACN evaporation. Once the plugs were dry, 50 μL of 10 mM DTT was added and tubes were incubated at 56°C for 15 min, for protein reduction. For alkylation, the remaining liquid was removed and 50 μL of 50 mM iodoacetamide (IAM) was added and the plugs were incubated in darkness for 60 min at RT. The digestion was carried out by adding 50 μL of freshly prepared 12.5 ng/μL trypsin (Roche, Basel, Switzerland) suspended in 50 mM ABC solution supplemented with 10% ACN and incubated at 37°C for 18 h. The resulting peptides were extracted first with nanopure water and twice with water–acetonitrile–formic acid solution (45/50/5) then concentrated to a minimal volume (~10 μL), and mixed with 30 μL with 5% acetonitrile in nanopure water containing 1% formic acid.

Peptide samples were then subjected to electrospray LC-MS/MS using a Finnigan™ LTQ-FT™ tandem mass spectrometer (Thermo Fisher Scientific, USA). The peptides were separated by reversed-phase chromatography on a Zorbax SB-300 C-18 column (150 mm × 300 μm, 5 μm particle size 300 Å pore size) (Agilent Technologies, CA, USA) eluting with an acetonitrile gradient in water containing 0.1% formic acid from 5 to 60% over 40 min using a Surveyor MS pump (Thermo Fisher Scientific, USA). The eluent entered the electrospray source at a flow rate of 5–6 μL.min^−1^ produced by splitting the Surveyor flow of 100 μL.min^−1^ with an UpChurch variable flow splitter. The mass spectrometer was operated in the positive ion mode with helium as the collision gas; the mass/charge range acquired was 300–2000 m/z. The capillary temperature was set at 210°C; the source voltage set at 3.8 kV. Data were acquired using a Top 5 experiment (one full scan in both the ion trap and ICR cell (parallel mode) followed by two averaged MS/MS microscans of each of the top five ions recorded in the ion trap) in data-dependent mode with dynamic exclusion enabled. Full scan Fourier transform data were obtained at a resolution of 100,000 at m/z 400 and used to refine the database search parameters.

Identification of the peptides was undertaken using Proteome Discoverer 1.4 (Thermo Fisher Scientific, USA), which was used to search fragment ion spectra matching peptides previously digested *in silico* using the proteome from maize (*Zea mays*) (http://www.plantgdb.org) and *T. virens* (http://genome.jgi-psf.org/TriviGv29_8_2) databases.

### Identification of apoplastic proteins by gel-free shotgun proteomics

For this analysis, APs (15 μg) for each biological replicate were digested separately. For each sample, 15 μL of 1 M of ABC were added, and adjusted to 100 μL with 50 mM ABC. Samples were reduced as follows: 1 M DTT was added to the samples to a final concentration of 10 mM, and then incubated at 56°C using 50 W for 15 min in a Discoverer II System microwave. The pH of the samples was adjusted to pH 8 with 1 M ABC. For alkylation, 1 M IAM was added to the samples to reach a final concentration of 50 mM. APs samples were kept in total darkness at RT for 30 min. After incubation, 1 M DTT was added to the samples to a final concentration of 20 mM. For protein digestion, 1 μg of trypsin (Promega, WI, USA) was added to 15 μg of total protein. Digestion was conducted for 60 min using microwave digestion at 45°C and 15 W power. After digestion, the reaction was quenched by addition of 2 μL of 50% formic acid. The tryptic peptides were desalted using a sensitive solid phase extraction (SPE) method with 1 mL Oasis HLB 10 mg extraction cartridges (Waters, MA, USA). Samples were diluted to 0.5 mL in 0.1% formic acid and pH was verified to a pH between 2 and 3 using pH indicator strips. A second SPE extraction was performed using 1 mL Oasis MCX 30 mg extraction cartridges (Waters, USA). APs samples were eluted with 0.3 mL of freshly prepared 50% acetonitrile in nanopure water, then concentrated in a Speedvac concentrator (Thermo Scientific, USA) to a volume between 10-15 μL, vortexed vigorously, then centrifuged at 16,000 × g for 30 s and finally diluted in 0.1% formic acid for MS analysis.

APs digests were separated on a 0.075 × 200 mm picofrit column (New Objective, Scientific Instrument Service, NJ, USA) packed with Reprosil C18 media (Dr Maisch GmbH HPLC, Entringen, Germany) using the following gradient: 0 min 5% B, 72 min 35% B, 76 min 95% B, 82 min 95% B, 83 min 5% B, 90 min 5% B, where A was 0.1% formic acid in water and B was 0.1% formic acid in acetonitrile. The gradient was formed at 250 nL/min using a NanoLC 400 UPLC system (Eksigent Technologies, CA, USA). The picofrit spray was directed into a TripleTOF 6600 Quadrupole-Time-of-Flight mass spectrometer (Sciex, MA, USA) scanning from 350 to 1600 m/z for 150 ms, followed by 40 ms. MS/MS scans on the 40 most abundant multiply-charged peptides (m/z 80–1600) for a total cycle time of 1.8 s. The mass spectrometer and HPLC system were under the control of Analyst TF 1.7 software (Sciex, USA).

The MS data were searched against a database which combined the Uniprot proteomes from *Z. mays* (http://www.uniprot.org/proteomes/UP000007305) and *T. virens* Gv29.8 (http://www.uniprot.org/proteomes/UP000007115) along with common contaminant entries (141,930 entries in total), using ProteinPilot version 5.0 (Sciex, USA) for peak picking identification selecting the following parameters: Cys-alkylation, iodoacetamide; digestion, full-trypsin digestion; and ID focus: biological modifications. Protein and peptide level false discovery rates (FDRs) were filtered to 1% and proteins with a threshold ProtScore ≤ 0.99 were discarded. The tandem MS/MS data was also analyzed using PEAKS Studio version 8 (BSI, ON, Canada). All resulting matched peptides were then confirmed by visual examination of the individual spectra.

### Label-free quantification analysis

The mass data was quantified by label free analysis using PEAKS software version 8 (BSI, Canada). Quantification was performed versus full tryptic digestion with a mass error tolerance of 20 ppm and 5.0 min for retention time shift tolerance. The quantification ratios were normalized using total ion current (TIC). Protein and peptide level FDRs were filtered to 1%. In addition, proteins with significance ≥ 10, fold change ≥ 1.5 and unique peptide ≥ 1 scores were selected.

### Identification of potential functional domains and gene ontology (Go) analysis

A comprehensive pipeline was designed to identify secreted proteins and predict their characteristic features such as: (a) presence of a signal peptide (SignalP 3.0 and 4.0) (Bendtsen et al., 2004b; Petersen et al., 2011), (b) presence of a transmembrane domains (TMHMM 2.0) (Emanuelsson et al., 2007); (c) subcellular localization (WolfPsort and TargetP 1.1) (Emanuelsson et al., 2000; Horton et al., 2007); and (d) non-classical protein secretion (SecretomeP 2.0) (Bendtsen et al., 2004a) (Supplementary Figure 3). Moreover, prediction of secretory proteins was based on bioinformatics tools and parameters reported by two different fungal secretome databases: Fungal Secretome Database (FSD) (Choi et al., 2010) and FunSeckB2 (Meinken et al., 2014), and one plant secretome database: PlanSeckB (Min et al., 2014). Additionally, the prediction tool EffectorP was used to identify putative effectors-like proteins from *T. virens* (Sperschneider et al., 2016). Predicted functional analysis of the APs was performed using Blast2GO that combines GO (http://geneontology.org/), BLAST (http://blast.ncbi.nlm.nih.gov/Blast.cgi), and Interpro (https://www.ebi.ac.uk/interpro) databases and searches for protein annotation (Supplementary Figure 3).

### Peroxidase activity

To determine peroxidase (POX) activity in the AF, the methodology described by Urbanek et al. (1991) was followed with modifications, using guaiacol as a hydrogen donor. The reaction mixture comprised 2 mL of a mixture containing 50 mM potassium phosphate buffer pH 6.8, 20 mM guaiacol (Sigma-Aldrich, USA) and 20 mM H_2_O_2_ (Merck Millipore, USA). The enzyme reaction was started by adding 10 μL containing 1 μg of APs and incubated for 10 min at 30°C. The reaction was stopped by adding 0.5 mL 5% (v/v) TCA and the absorbance was read at 480 nm. One unit of peroxidase activity was defined as the amount of enzyme that increased the absorbance by 0.01 expressed as units of POX/μg protein.

### Statistical analysis

Statistical analyses were performed using general analysis of variance (ANOVA) in the GenStat 18th package (VSN International, United Kingdom). Each value is the mean ± STD for 3 replicates in each group, and *P* ≤ 0.05 was considered as significant.

## Results

### *Trichoderma virens*-maize root interaction

**Figure 1:**
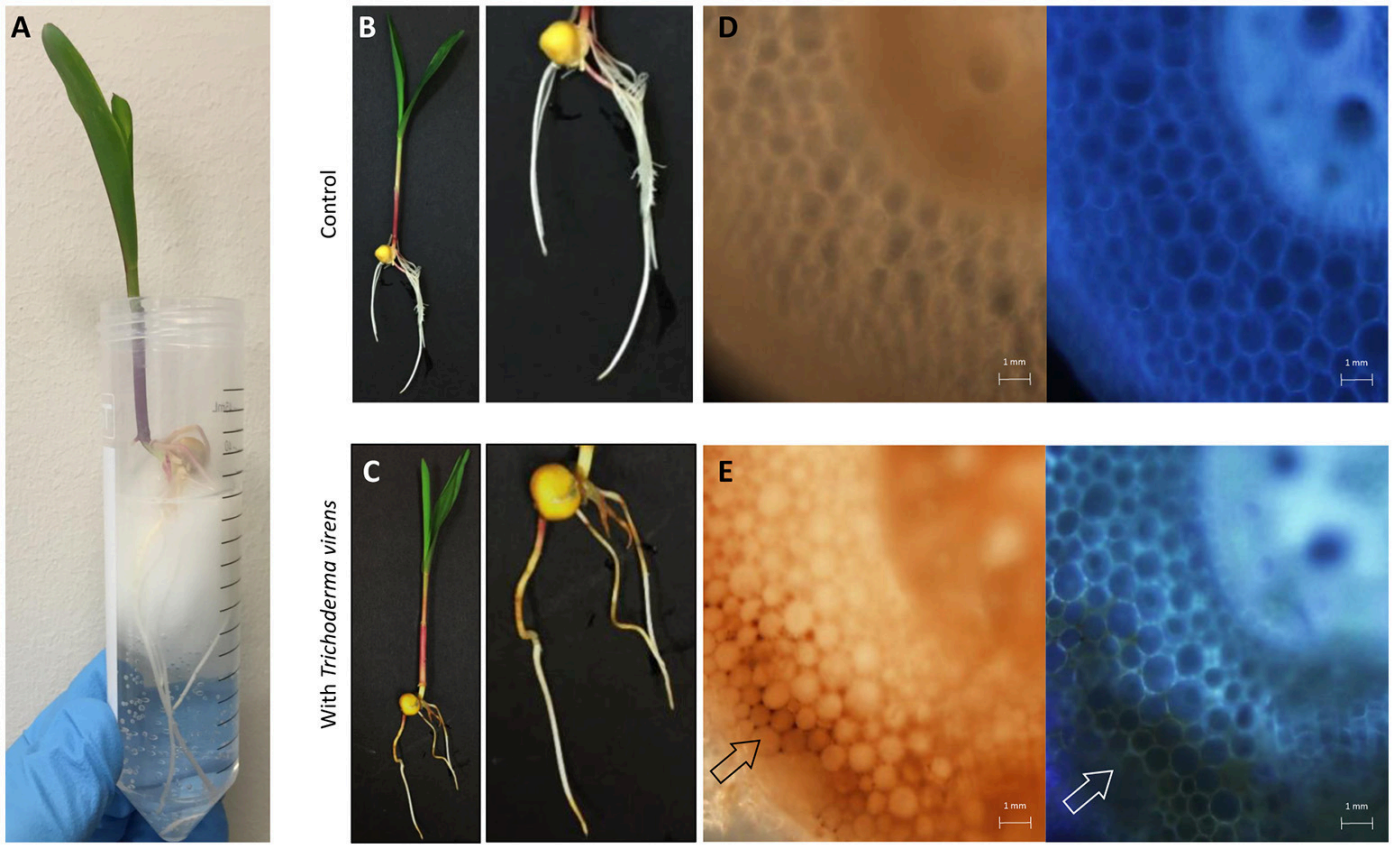
Overview of *T. virens*-maize interaction under a hydroponic system. **(A)** Five day old maize seedling growing aseptically under hydroponic conditions. **(B)** Un-inoculated and **(C)** inoculated plants with *T. virens*. Inoculated plants show phenotypical changes in their root system compared to the control. Cross section of un-inoculated primary root **(D)** bright-field and DAPI. Cross section of inoculated primary root **(E)** showing accumulation of brown pigmentation in epidermal and cortical cells, bright-field and DAPI. Images were obtained with a fluorescent microscope.

A hydroponic system was used to assess communication between *T. virens* and maize plant roots (Figure 1A). When maize seeds were inoculated with the fungal spores and then germinated in germination paper soaked with Hoagland's solution, germination was not substantially inhibited by the fungus (data not shown).

The presence of *T. virens* altered seedling root morphology when compared with un-inoculated seedlings (Figures 1B,C); a reduction in secondary root length and the presence of a brownish color on the surface where evident in the inoculated seedlings (Figures 1C,E). The brownish color was independent of the original *T. virens* spores inoculum (seedlings inoculated from 10^4^ to 10^7^ spores per seed, Supplementary Figure 1). This pigmentation extended from epidermal to cortical cell layers compared to un-inoculated roots (Figures 1D,E), but was not observed in the vascular system (Figure 1E).

**Figure 2:**
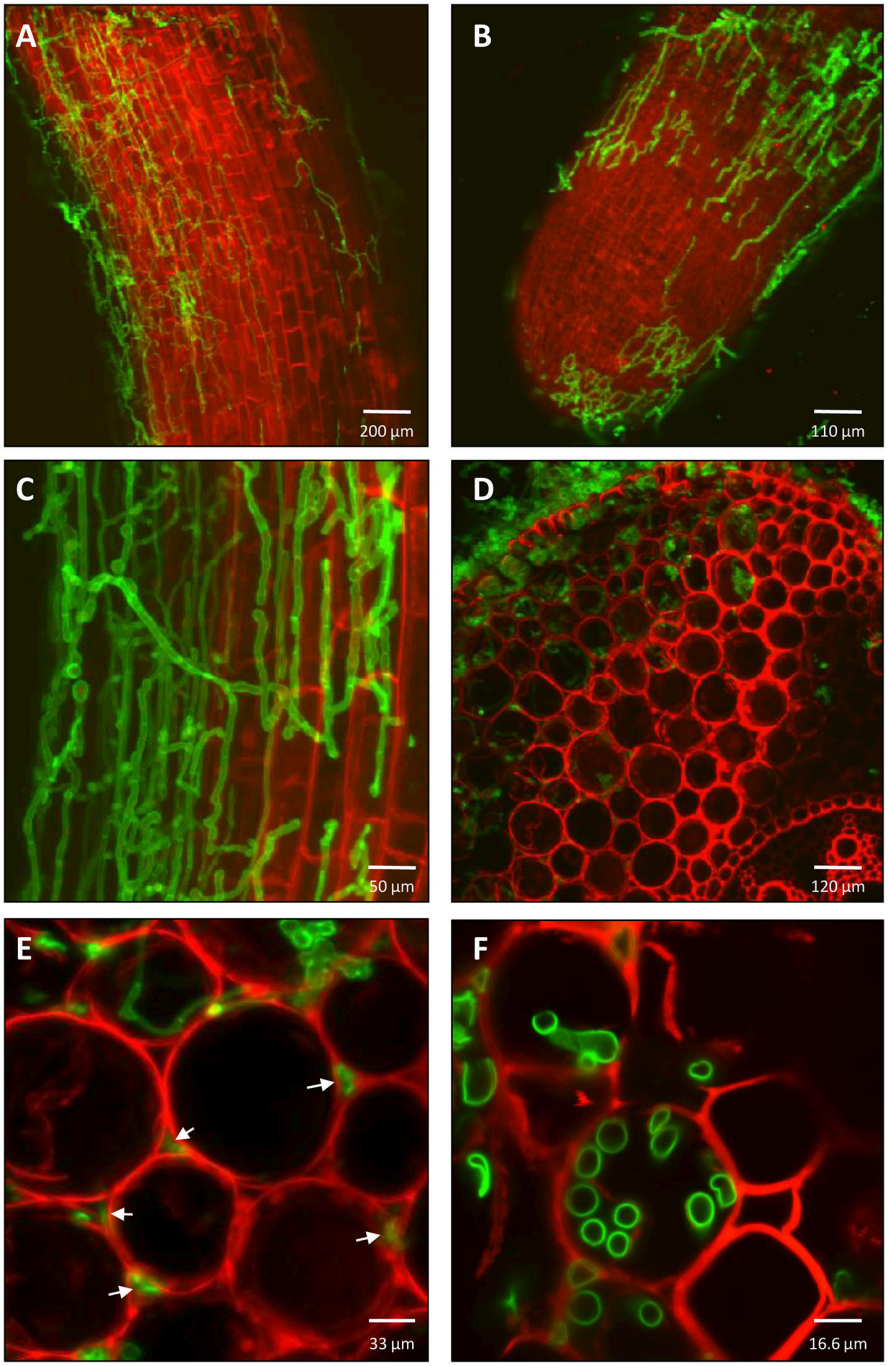
Colonization pattern of *T. virens* in maize roots. **(A)** After 5 days post inoculation, *T. virens* hyphae inhabit epidemical cells of maize primary root and **(B)** root tip of secondary root. **(C)** Close up of the hyphae occupying epidemical cells of the differentiation zone of the primary roots. **(D)** Cross section of primary root showing internal colonization of epidermal and cortical layers near to vascular system. **(E)** Intercellular and **(F)** intracellular colonization of cortex cells by *T. virens* hyphae (arrows). Fungal and plant cells were detected using WGA-Alexa Fluor 488 (green channel), propidium iodide (PI) and FM 4-64 Dye (red channel). Plant cell walls were detected with PI **(A-F)** and plant plasma membrane with FM 4-64 **(D-F)**. Fungal cells were detected with WGA-Alexa Fluor 488 **(A-F)**. Images were obtained with a confocal microscope.

To examine the colonization of *T. virens* in the root system, dual staining of the fungal cell wall (WGA- Alexa Fluor™ 488) and the plant cell wall (PI) or plant membrane (FM 4-64) was performed. Superficial fungal root colonization was predominantly found in the first two centimeters of the primary root (close to the seed) compared with other sections of the primary root (Figures 2A,C). In addition, *T. virens* colonized secondary roots and new root tips (Figure 2B). A transverse cut of the primary maize root enabled visualization of the internal colonization by *T. virens*. The fungus colonized the cortex layer adjacent to the vascular system of the primary root (Figure 2D). *T. virens* colonized intercellular spaces (apoplast) (Figure 2E). To visualize if *T. virens* colonized intracellular spaces, we used the plasma membrane dye (FM 4-64) in combination with WGA- Alexa Fluor™ 488 to follow the fungus. As observed in Figure 2F, *T. virens* colonized intracellular spaces and grew between the plasma membrane and the plant cell wall.

### Isolation and identification of apoplastic proteins from maize root seedlings confronted or not with *T. virens* Gv29.8

To determine the efficiency of different methodologies used for the extraction of AF from maize roots, two methods were tested. APs were successfully obtained by the infiltration-centrifugation system compared to the sorption when observed on the 1-D SDS-PAGE gel (Supplementary Figures 2A,B).

To evaluate levels of cytoplasmic contamination the activity of malate dehydrogenase (MDH) was used as a biomarker, which is commonly tested during extraction of AF (Gupta et al., 2015). The activity of MDH detected in AF extracted from un-inoculated and inoculated roots was up to 1.5% compared to the total soluble protein extract from roots (Supplementary Table 1). MDH levels below 2% are considered suitable for plant apoplast studies (Dannel et al., 1995; Dragišić Maksimović et al., 2008, 2014; Yang et al., 2015).

Multiple methods were used to obtain proteome coverage; in this study we compared Gel-LC-MS/MS and gel-free shotgun proteomics.

#### Identification of apoplastic proteins by Gel-LC-MS/MS

APs were separated by 1-D SDS-PAGE, with three biological replicates for each condition (Supplementary Figure 2A). The APs showed differences in their protein complement with distinctly different protein bands visible in inoculated plants (M+Tv) compared with un-inoculated plants (M). The patterns of protein fractions from 15 to 75 kDa and 130 to 250 kDa were largely similar in their intensity and mass separation. Differences were observed in the protein fractions between 75 and 130 kDa sections in inoculated samples compared with the un-inoculated (Supplementary Figure 2A). Specifically, two treatment-specific bands were observed; one was located above the 100 kDa fraction and the other below (Supplementary Figure 2A). Based on their mass, the protein bands were cut from the gels in five sections (Supplementary Figure 2A), and then analyzed by LC-MS/MS.

Using this gel-based proteomic approach coupled with LC-MS/MS, 13 proteins were identified, of which 12 corresponded to maize and one to *T. virens*. Five of these maize proteins: LRR receptor-like serine/threonine-protein kinase (A0A1D6ERY2_MAIZE), aspartic-type endopeptidase (A0A1D6F8J3_MAIZE), germin-like protein subfamily T member 1 (B4FRS8_MAIZE), barwin-like protein (Win1) (B6SH12_MAIZE), and peroxidase (Per66) (PER66_MAIZE) were common to both conditions (inoculated and un-inoculated maize samples) and were located in the 75–15 kDa fractions. Proteins that were only present in the un-inoculated samples included two peroxidases (C0PGF4_MAIZE; A0A1D6PD14_MAIZE) and a pathogenesis-related protein 1 (PR-1) (A0A1D6K5Y8_MAIZE) which were identified in the 50–15 kDa fractions. Four maize proteins identified from the inoculated samples exclusively were in the 120–30 kDa fractions: methionine synthase (Q8W529_MAIZE), heat shock protein 70 (A0A1D6MWU7_MAIZE), adenosylhomocysteinase (A0A1D6PTE3_MAIZE), and pectinesterase (B6SSX0_MAIZE). In contrast, the protein TV_29366, which encodes for a β-xylosidase, was the only protein detected from *T. virens* and this was present in the 120–75 kDa fractions.

#### Identification of proteins by Gel-free shotgun proteomics

The low number of proteins identified by Gel-LC-MS/MS base technology drove us to use a more powerful proteomic tool (gel-free shotgun proteomics) to increase the identification number of proteins present in the apoplast which has a complex protein mixture. Using the gel-free shotgun proteomics approach, 148 maize proteins were identified in the un-inoculated control roots that were present in all three biological replicates. In the inoculated roots, a total of 177 were identified, where 85 and 92 proteins corresponded to the maize and *T. virens* proteomes, respectively.

Interestingly, in comparison with the un-inoculated roots, the inoculated roots showed a 43% (63 proteins) reduction in the number of total maize proteins identified. These results show that an alteration in the maize proteome is triggered by the presence of *T. virens*. In contrast to Gel-LC-MS/MS, gel-free shotgun proteomics showed an increase of identified proteins from 13 to 272, showing that gel-free shotgun proteomics coupled with next generation LC-MS/MS instruments, is a useful technology to identify larger numbers of proteins in complex samples, such as in AF during plant-microbe interactions.

### Prediction and annotation of secreted apoplastic proteins through bioinformatics tools

Putative secreted proteins were classified into two classes: (a) classical secreted proteins and (b) non-classical secreted proteins. In addition, the literature was used as a point of reference for those proteins that were not predicted as being derived from classical and non-classical secretion systems by bioinformatics analysis, but have been reported as secreted proteins in other fungal and plant models. Based on the above criteria, in the un-inoculated maize roots 76 (51%) of the proteins were predicted to be secreted. Of these 56 (74%) followed the classical secretion system, while 20 (26%) were seemingly secreted by a non-classical mechanism and consequently were classified as leaderless secreted proteins (LSPs) (Supplementary Figure 4A). From the 85 maize proteins identified from the inoculated maize roots, 49 were predicted to be secreted and of these, 22 (45%) followed the classical and 27 (55%) the non-classical secretion system (Supplementary Figure 4A). In addition, un-inoculated roots showed 46 unique secreted proteins, while 18 unique secreted proteins were present in inoculated roots (Supplementary Figure 4A). Of 92 conserved *T. virens* proteins, 43 (46%) were predicted to be secreted, where 20 (46%) followed the Golgi-ER secretion system and 23 (54%) were secreted by non-conventional secretion systems (Supplementary Figure 4B). These results indicate that both organisms use both classical and non-classical secretion systems to deliver proteins into the apoplast.

### Functional annotation of maize secreted apoplastic proteins at 5 days interaction

**Figure 3:**
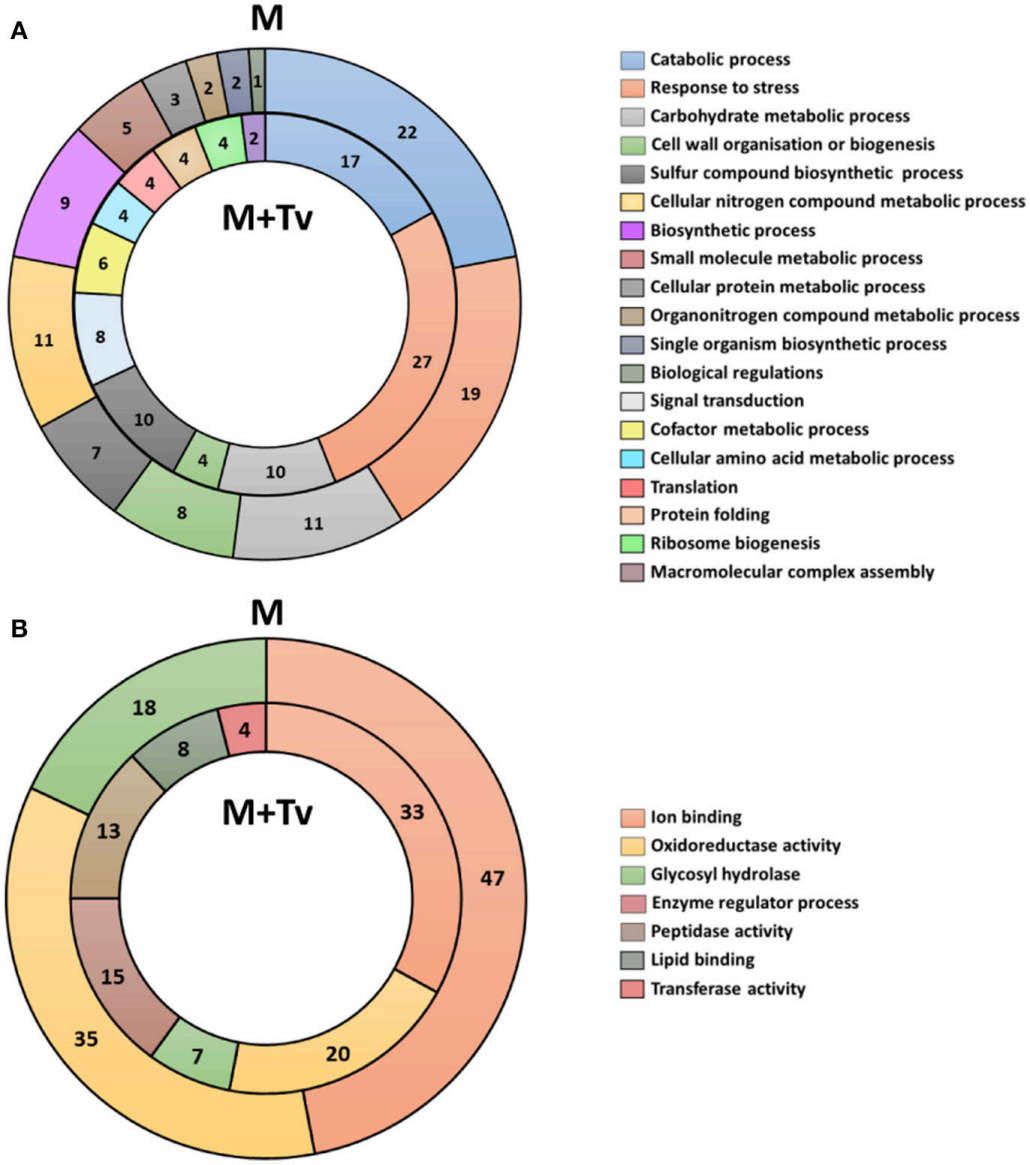
Functional classification of all secreted proteins from un-inoculated and inoculated maize roots at 5 days interaction. **(A)** Blast2GO multilevel chart for biological process of un-inoculated (M) and inoculated (M+Tv) maize roots. **(B)** Multilevel chart for molecular function of un-inoculated and inoculated maize roots. Score distribution represented as a percentage of each group is indicated inside the pie slices.

Secreted proteins were organized into functional categories for biological processes and molecular function based on their gene ontologies (GO) (Figures 3A,B). In un-inoculated maize roots, the four major biological process groups of proteins were catabolic processes (22%), response to stress (19%), carbohydrate metabolic processes (11%) (Figure 3A), and cellular nitrogen compound metabolic process (11%). The three main molecular functions were ion binding (47%), oxidoreductase activity (35%) and glycosyl hydrolase activity (18%) (Figure 3B). By comparison in inoculated samples, the four major biological process functional groups were response to stress (27%), catabolic processes (17%), carbohydrate metabolic processes (10%), or sulfur compound biosynthetic processes (10%) (Figure 3A). The three main molecular functions were ion binding (33%), oxidoreductase activity (20%) and enzyme regulator activity (15%) (Figure 3B).

Multiple changes were identified in the predicted suite of functions of the proteins secreted from un-inoculated compared with inoculated roots. Response to stress was increased during the interaction with *T. virens* from 19 to 27%; in contrast, catabolic processes were reduced from 22 to 17%. No differences were observed in proteins belonging to carbohydrate metabolic processes; however, some differences were present in secondary metabolism, changing from nitrogen to sulfur metabolic processes (Figure 3A). Major changes in molecular functions were observed; a reduction of ion binding from 47 to 33%, oxidoreductases from 35 to 20%, and glycosyl hydrolases from 18 to 7% activity. Conversely, lipid binding, protease and transferase activities were present only in inoculated roots (Figure 3B).

Putative identification based on conserved domains and function was performed on the identified protein family members from maize roots that were secreted into the apoplast in un-inoculated and inoculated plants after 5 d interaction. The major protein groups from maize were: (a) 11 glycosyl hydrolases (GHs) that are involved in the degradation of carbohydrate complexes; (b) 9 antioxidant proteins that catalyze reactions to neutralize free radicals and ROS; (c) 15 peroxidases, that participate in the biosynthesis of the cell wall and defense responses and have multiple tissue-specific functions; (d) pathogenesis-related (PR) family proteins that are activated under biotic stresses; (e) proteases/peptidases that are responsible for the hydrolysis of peptide bonds, and (f) proteinase inhibitors (PIs) that participate in the inactivation of proteases. Additionally, other family groups that were identified belonged to DUF proteins, oxidoreductases, lipases, ribonucleases, chaperones, calmodulin, ribosomal proteins and cyclophilin (Table 1 and Supplementary Table 2).

**Table 1:** Summary of the apoplastic proteins secreted by *Zea mays* after 5 days interaction (inoculated).

**Uniprot ID^a^**	**Protein annotation^b^**	**Protein group**
GSTF4_MAIZE	Glutathione S-transferase (Gst4)^*^^3^	Detoxifying and ROS related enzymes
B6TL20_MAIZE	Glutathione S-transferase (Gstu6)	
C0PK05_MAIZE	Lactoylglutathione lyase^*^^4^	
A0A1D6QGI0_MAIZE	Peroxidase (Per67)	
A0A1D6N0K3_MAIZE	Peroxidase (Per12)	
K7VH58_MAIZE	Peroxidase (Per52)	
A0A1D6F4C8_MAIZE	Peroxidase (Per66)	
A0A1D6KAW3_MAIZE	Peroxidase (Per54)	
A0A1D6E530_MAIZE	Peroxidase (Per12)	
SODC5_MAIZE	Superoxide dismutase [Cu-Zn]	
B6SH12_MAIZE	Barwin superfamily protein (Win1)	Microbe related proteins
Q9SYS1_MAIZE	Beta-amylase (Amy2)	
B4FTS6_MAIZE	Endochitinase A	
C0P451_MAIZE	Chitinase B1	
B4FWD0_MAIZE	Minor allergen Alt a7	
B6TFN1_MAIZE	Minor allergen Alt a7	
A0A1D6JZU3_MAIZE	Pathogenesis-related protein 10 (PR10)^*^^5^	
Q29SB6_MAIZE	Pathogenesis-related protein 10 (PR10)^*^^5^	
A0A1D6N932_MAIZE	Osmotin-like protein OSM34	
A0A1D6GKZ3_MAIZE	Osmotin-like protein OSM34	
A0A0B4J327_MAIZE	Aspartic-type endopeptidase	Proteases
A0A096RR58_MAIZE	Cysteine-type endopeptidase	
C0PBS1_MAIZE	Lipase	Protein involved in lipid metabolism
B6SHR9_MAIZE	PVR3-like protein	
Q7FU57_MAIZE	Bowman-Birk type wound-induced proteinase inhibitor (Wip1)	Proteinase inhibitors
Q42420_MAIZE	Proteinase inhibitor (Pis7)	
C0HII8_MAIZE	Bowman-Birk type trypsin inhibitor	
K7U234_MAIZE	Serine-type endopeptidase inhibitor	
B6SNA6_MAIZE	Subtilisin-chymotrypsin inhibitor CI-1B	
B4FBW7_MAIZE	Calmodulin	Proteins involved in signaling
B6SIF5_MAIZE	Translationally-controlled tumor 1 protein	
C0P4M0_MAIZE	Monodehydroascorbate reductase 1 peroxisomal	Proteins involved in cell redox homeostasis
Q5EUE1_MAIZE	Protein disulfide-isomerase (Pdil1-1)	
C0HGV5_MAIZE	Enolase 2 (Eno2)^*^^1^^,^^3^^,^^6^^,^^7^	Proteins involved in energy production pathways
SCRK1_MAIZE	Fructokinase-1 (Frk1)^*^^2^	
B4FAG0_MAIZE	GDP-mannose 4,6 dehydratase^*^^9^	
A0A1D6HV20_MAIZE	Glucose/Sorbosone dehydrogenase	
Q8S4W9_MAIZE	Pyruvate decarboxylase (Pdc3)	
B6T1H5_MAIZE	60S ribosomal protein L12	Proteins involved in protein synthesis, folding and stabilization
B7ZZ42_MAIZE	Heat shock 70 protein^*^^3^	
B4FZZ2_MAIZE	Peptidyl-prolyl cis-trans isomerase (Cyclophilin)^*^^1^^,^^7^	
K7UR51_MAIZE	40S ribosomal protein S8	
A0A1D6N1Z8_MAIZE	6-phosphogluconate dehydrogenase	Proteins involved in secondary metabolism
C0PHR4_MAIZE	Adenosylhomocysteinase^*^^1^^,^^3^	
BX9_MAIZE	DIMBOA UDP-glucosyltranferase (BX9)^*^^8^	
C0PEP2_MAIZE	Acc oxidase 1^*^^6^	
C0P5Y3_MAIZE	Methionine synthase^*^^3^^,^^6^	
PROF5_MAIZE	Profilin-5	Structural protein
A0A1D6E7A7_MAIZE	DUF642 protein	Unknown

a www.uniprot.org/uniprot/;

b Blast2GO https://www.blast2go.com/;

* Hypothetical secreted proteins identified by literature review:

1 Tanveer et al., 2014;

2 Hajirezaei et al., 2000;

3 Agrawal et al., 2010;

4 Zhang et al., 2016;

5 Choi et al., 2012;

6 Ding et al., 2012;

7 Fernández et al., 2012;

8 Schulz et al., 2016;

9 *Liao et al., 2012*.

Interestingly, protein family groups were founded in higher abundance in un-inoculated compared to inoculated roots, for example, maize glycosyl hydrolases (GHs) were reduced from 15 to 6%, respectively. Similar results were identified in the peroxidase group where their reduction was from 19 to 13%. In contrast, pathogen-related proteins increased from 3 to 10% and protease/peptidase and PIs from 8 to 16 %. These results indicate that the maize proteome is altered by the presence of *T. virens* by the expression and suppression of different protein families, suggesting that the fungus is re-shaping the plant secretome.

### Functional annotation of *T. virens* secreted apoplastic proteins at 5 days interaction

**Figure 4:**
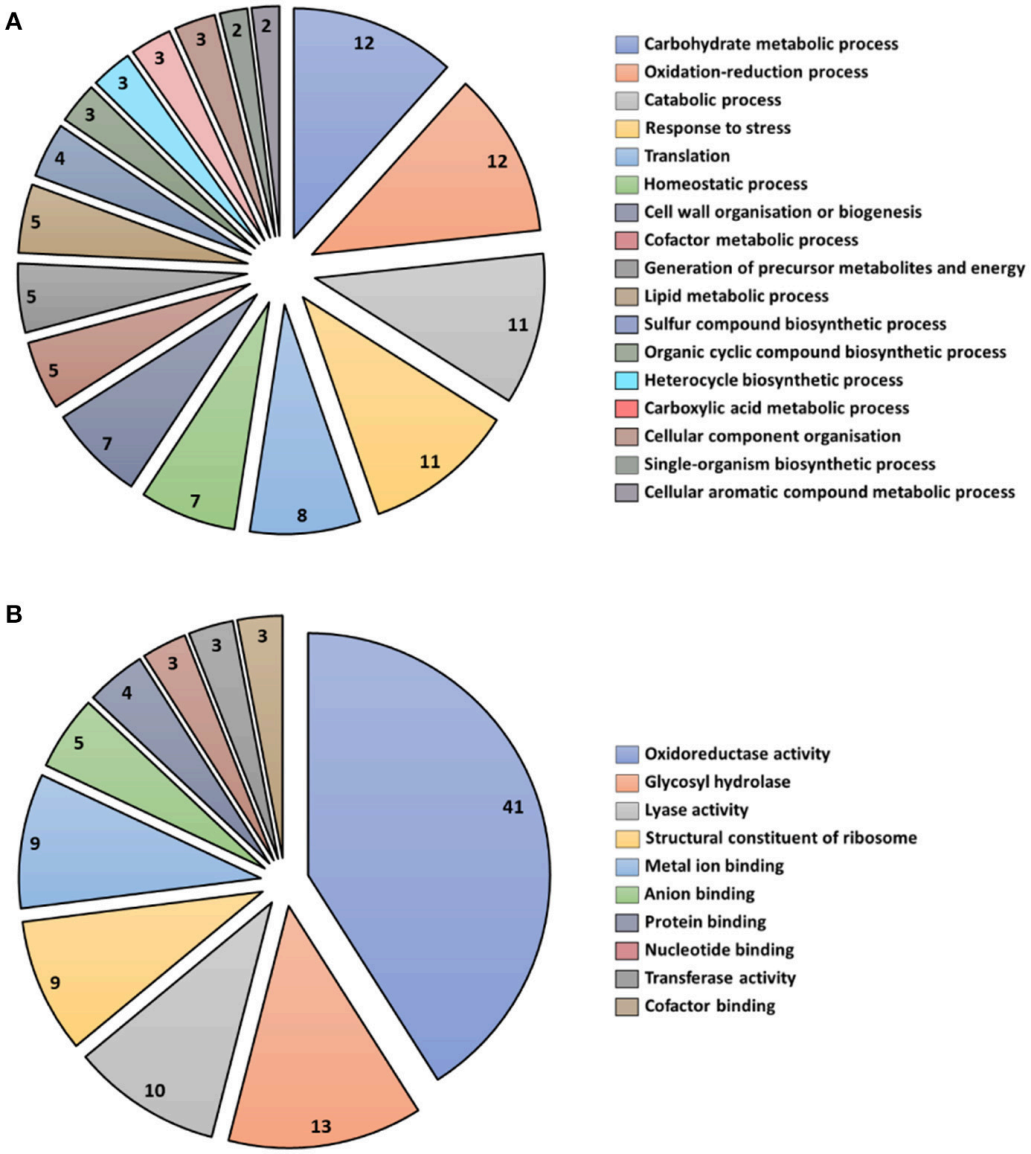
Functional classification of all secreted proteins from *T. virens* at 5 days interaction. Blast2GO multilevel chart for **(A)** biological process and **(B)** molecular function. Score distribution represented as a percentage of each group is indicated inside the pie slices.

A total of 43 secreted proteins from *T. virens* were identified during the interaction with maize roots (Table 2). Secreted proteins were organized into functional categories for biological processes and molecular function based on their gene ontologies (GO) (Figures 4A,B). The four major biological process groups of proteins identified were: carbohydrate metabolic process (12%), oxidation-reduction process (12%), catabolic process (11%), and response to stress (11%) (Figure 4A). The three main molecular functions were oxidoreductase activity (41%), glycosyl hydrolase (13%) and lyase activity (10%) (Figure 4B).

**Table 2:** Summary of the apoplastic proteins secreted by *Trichoderma virens* after 5 days interaction.

**Protein Identifier**		**Protein putative-annotation^c^**	**Protein group**
**JPGI^a^**	**Uniprot ID^b^**		
TV_75509	G9N8W5_HYPVG	Enolase^*^^5^^,^^7^	Proteins involved in energy producing pathways
TV_87809	G9N9Z6_HYPVG	Galactose mutarotase-like protein	
TV_92614	G9N6G5_HYPVG	Malate dehydrogenase^*^^8^^,^^9^^,^^10^	
TV_42143	G9N7I4_HYPVG	Beta-galactosidase	Glycoside hydrolases
TV_78372	G9MWK2_HYPVG	Beta-galactosidase	
TV_90504	G9MY26_HYPVG	Cellobiohydrolase	
TV_110754	G9ML80_HYPVG	ß-glycosidase	
TV_29366	G9MSH9_HYPVG	ß-xylosidase	
TV_71600	G9MLE1_HYPVG	-glycosidase	
TV_50666	G9N1W4_HYPVG	Cupin 1/Bicupin	Microbial elicitor
TV_110852	G9MJD8_HYPVG	Small protein 1 (Sm1)	Small secreted protein
TV_111995	G9MUU9_HYPVG	SSCP	
TV_92810	G9N192_HYPVG	SSCP/CFEM	
TV_111061	G9MI10_HYPVG	Protein disulfide-isomerase (pdi1)	Proteins involved in protein synthesis, folding and stabilization
TV_138628	G9MTV5_HYPVG	Pyridine nucleotide-disulphide oxidoreductase	
TV_139551	G9N846_HYPVG	Ribosomal protein 60S	
TV_78230	G9NDN5_HYPVG	Ribosomal protein L11C	
TV_88756	G9N6G6_HYPVG	Ribosomal protein L28e	
TV_74544	G9NCR5_HYPVG	Ribosomal protein S7	
TV_216375	G9MYT5_HYPVG	Proteinase inhibitor	Proteinase inhibitor
TV_58449	G9N458_HYPVG	Glucose-methanol-choline oxidoreductase	Protein involved in cell redox
TV_53497	G9MU78_HYPVG	Aminotransferase (GliI)^*^^11^	Proteins involved in secondary metabolism
TV_82877	G9N875_HYPVG	S-adenosylhomocystein hydrolase ^*^^3^^,^^4^	
TV_215323	G9MGG3_HYPVG	Alcohol dehydrogenase	
TV_74949	G9NAQ0_HYPVG	Cytochrome P450	
TV_186579	G9MVE5_HYPVG	Cytochrome P450^*^^12^	
TV_87758	G9NA55_HYPVG	S-adenosylmethionine synthase^*^^4^	
TV_72386	G9MID9_HYPVG	Short-chain dehydrogenase/reductase (SDR)	
TV_91355	G9MU80_HYPVG	S-adenosyl-L-methionine methyltransferase (GliN)^*^^11^	
TV_215037	G9MF42_HYPVG	Thiamine biosynthesis protein (Thi4)	
TV_88738	G9N6E1_HYPVG	Translation controlled tumor-associated (TCTP)	Proteins involved in signaling processes
TV_217216	G9NCG7_HYPVG	14-3-3 protein^*^^6^	
TV_215514	G9MJV5_HYPVG	Catalase-peroxidase haem^*^^1^^,^^6^^,^^10^	Proteins involved in stress/defence mechanisms
TV_54541	G9MKH2_HYPVG	Glutathione reductase^*^^4^	
TV_72131	G9ML11_HYPVG	Heat shock protein Hsp70 (bip1)	
TV_72615	G9MJ35_HYPVG	L-domain-like protein (Ecm33)	
TV_183329	G9N7N4_HYPVG	Superoxide dismutase [Cu-Zn]	
TV_81963	G9MIH3_HYPVG	Thioredoxin reductase^*^^2^	
TV_216458	G9N026_HYPVG	Thioredoxin-related protein	
TV_40034	G9NAK1_HYPVG	Hypothetical protein	Unknown
TV_76398	G9N4X8_HYPVG	Hypothetical protein (HGD-D superfamily)	
TV_141673	G9MH43_HYPVG	Hypothetical protein DUF3759 (CipC1)	
TV_216138	G9MTV6_HYPVG	Hypothetical protein DUF1857^*^^6^	

a http://genome.jgi.doe.gov/TriviGv29_8_2/TriviGv29_8_2.home.html;

b www.uniprot.org/uniprot/;

c Blast2GO https://www.blast2go.com;

* Hypothetical secreted proteins identified by literature review:

1 Tanabe et al., 2011;

2 Shi et al., 2012;

3 Luo et al., 2008;

4 Lamdan et al., 2015;

5 López-Villar et al., 2006;

6 Yang et al., 2015;

7 Sundstrom and Aliaga, 1994;

8 Giardina and Chiang, 2013;

9 Weber et al., 2012

10 Chu et al., 2015;

11 Kim et al., 2014;

12 *Druzhinina et al., 2012*.

Identification of putative proteins based on their conserved domains and function was performed on the proteins from *T. virens* that were secreted into the apoplast in inoculated plants after 5 d interaction. The glycosyl hydrolase (GHs) family was the highly represented in the *T. virens* secretome (Table 2). Proteins that participate in antioxidant processes, oxidate stress resistance and secondary metabolism were also identified (Table 2). Furthermore, in the presence of maize roots, groups of proteins corresponding to putative effector-like proteins, chaperones, 14-3-3 like proteins and ribosomal proteins were identified as part of the *T. virens* secretome (Table 2). Overall, these results suggest that *T. virens* activates different molecular mechanisms during host root colonization.

### Label-free quantification of apoplastic proteins during the *T. virens*-maize interaction

#### Label-free quantification

By using the label-free quantification approach we identified 10 proteins from maize that were significantly (significance ≥10; fold change ≥1.5) different in their intensities between un-inoculated and inoculated roots (Supplementary Figure 5). Analysis of the correlation of the signal intensities showed that the biological repeats of each treatment were clustered together, with an average correlation of 0.87 for un-inoculated roots (M) and 0.90 for inoculated roots (M+Tv). Correlation between all biological repeats showed an average relationship of ≥0.70. Proteins that showed a decreased abundance during the interaction with *T. virens* were 40S ribosomal protein (B4FSW0_MAIZE), pathogenesis-related protein (PR-10) (Q29SB6_MAIZE), peroxidase (Per12) (B4FG39_MAIZE), cytosolic ascorbate peroxidase (Apx1) (B6TM55_MAIZE), cysteine endopeptidase (K7W288), blue copper protein (B6UHQ8_MAIZE), adenosylhomocysteinase (C0PHR4_MAIZE), protein disulphide-isomerase (Pdil1-1) (Q5EUE1_MAIZE), and ribonuclease 1 (B4FBD6_MAIZE). In contrast, the protein serine-type endopeptidase inhibitor (K7U234_MAIZE) showed an increased abundance. Overall, these results suggest that the abundance of proteins involved in different plant biological processes, including plant defense, are manipulated by the presence of *T. virens*.

### Peroxidase levels influenced by *T. virens*

**Figure 5:**
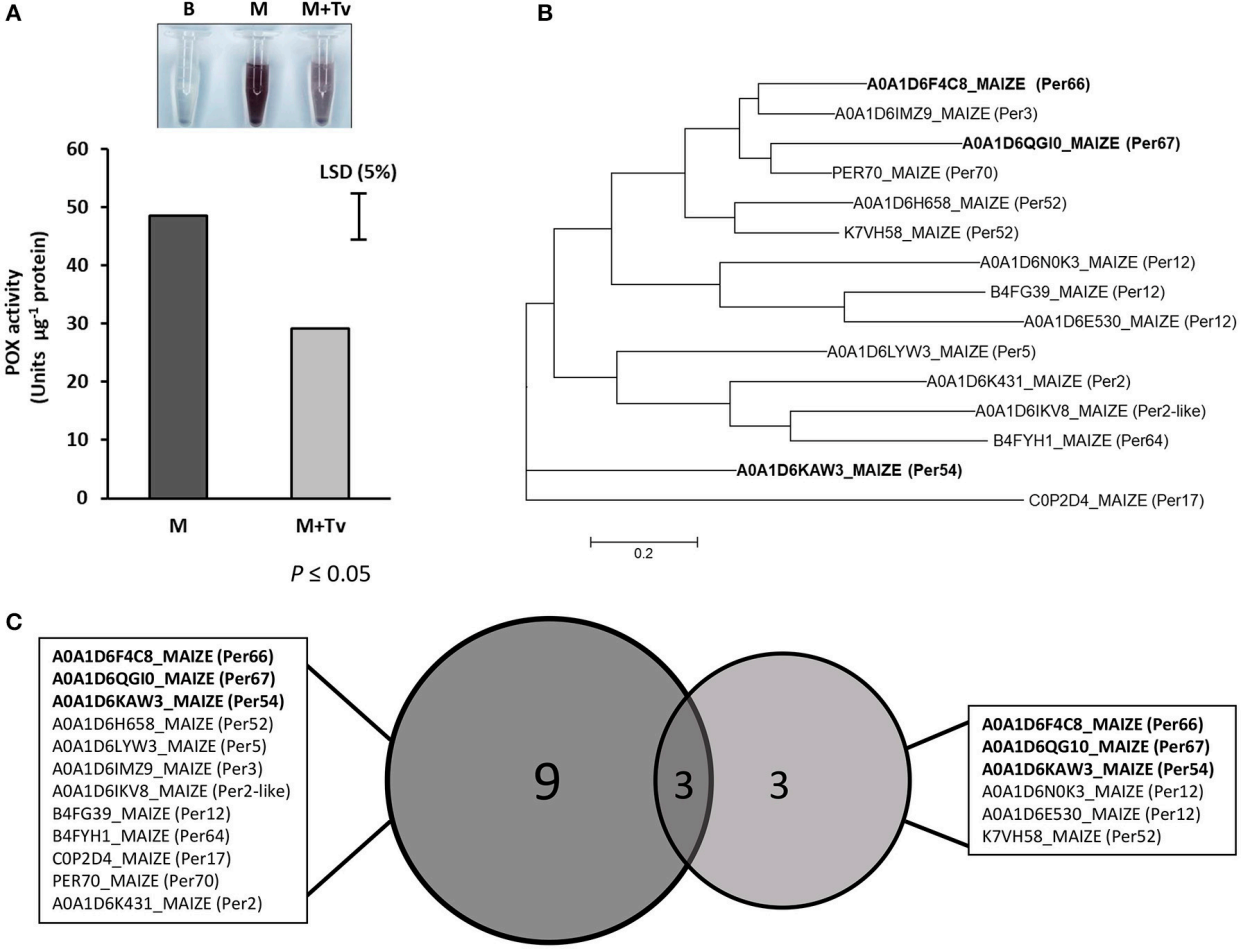
Peroxidase study during *T. virens*-maize interaction. **(A)** Peroxidase activity in un-inoculated (M) and inoculated (M+Tv) maize roots with *T. virens* after 5 days interaction (*p* ≤ 0.05). **(B)** Phylogenetic tree of peroxidases identified in the maize apoplast zone. Using Muscle, the composite proteins were aligned, and the Maximum likelihood tree, were generated in Mega 6. **(C)** Comparison between peroxidases expressed in maize roots with or without *T. virens*. Bold names represent peroxidases that were present in both conditions.

The peroxidase activity in the AF after 5 d interaction was measured in maize root tissues in un-inoculated and inoculated with *T. virens*. Shifts in the enzyme activity were observed, where inoculated roots with *T. virens* showed a significant reduction in peroxidase activity compared with un-inoculated roots (*p* ≤ 0.05) (Figure 5). These results indicated that the peroxidase activity was higher in un-inoculated roots compared to inoculated, showing that the peroxidase activity was directly influenced by the presence of *T. virens* in the root system.

## Discussion

In this study, we observed the interaction between maize roots and *T. virens* by confocal microscopy. In addition, we identified the secretome profile of maize roots growing alone or during the interaction with *T. virens*. Furthermore, the identification and function of these proteins were analyzed to understand the molecular dialogue that exists in the apoplast between *T. virens* and maize, and unravel the role that these proteins may play during the symbiotic interaction.

### Root interaction with *T. virens*

Previous reports elucidate the lifestyle of *T. virens* as endophytic in different host plants (Vargas et al., 2009; Moran-Diez et al., 2015; Lawry, 2016; Romão-Dumaresq et al., 2016). Our findings showed that *T. virens* colonized different sections of the root system, including primary and secondary roots (Figure 2). Interestingly, *T. virens* is able to endophytically colonize inter- and intracellular spaces of maize roots (Figure 2), suggesting that the fungus utilizes both pathways to establish itself in the host root system.

During the interaction, phenotypic responses of maize roots were detected when colonized by *T. virens*, for example, appearance of a brownish pigment and reduction of secondary root growth (Figure 1). Accumulation of browning of inoculated roots has been observed previously in *T. virens* (Moran-Diez et al., 2015) and *Trichoderma harzianum* (Palaniyandi et al., 2017) and in other fungal systems, including incompatible interaction of arbuscular mycorrhizal fungi with *Salsola kali* (Allen et al., 1989) or in detrimental interactions of pathogens such as *Pythium aphanidermatum* or *Fusarium graminearum* with their host roots (Sutton et al., 2006; Ye et al., 2013). Therefore, it could be argued that maize cells are responding to *T. virens* colonization by triggering the accumulation of phenolic compounds involved in initial responses to stress to reinforce plant cell walls and inhibit fungal growth (Beckman, 2000). Nevertheless, it cannot be discounted that the physiological changes on maize roots were due to the deposition of *T. virens* secondary metabolites such as melanin onto the root surface or into the media or that *T. virens* mycelia were blocking the aeration of the media creating anoxic conditions and inducing this physiological change (browning).

Endophytic colonization (both inter- and intracellular) by *T. virens* showed different mechanisms that the fungus undertakes to develop an interaction with its host plant and to promote fungal growth on plant tissue. A similar pattern of colonization was observed in the endophytic fungus *Piriformospora indica* interaction with barley plants (Deshmukh et al., 2006). This symbiotic fungus requires host cell death in differentiated barley roots, in order to proliferate and become endophytic and form a mutualistic interaction. This implies that the fungus biotrophically colonizes by digesting plant cell walls and subsequently either interferes with the host cell death program or actively kills host cells (Deshmukh et al., 2006). We suggest that under hydroponic conditions *T. virens* may employ similar mechanisms to colonize maize roots. Therefore, our interest was to elucidate the molecular mechanisms that occur in the apoplastic zone for developing an endophytic relationship by the manipulation of host defense responses.

### Identification of apoplastic proteins by gel-based proteomic technology

The infiltration-centrifugation method was the most efficient approach for the extraction of AF and APs, which has been previously reported in other studies for the identification of microbe-secreted proteins *in planta* (Floerl et al., 2012; Shenton et al., 2012). Using the gel-based LC-MS/MS approach, maize proteins such as methionine synthase, heat shock protein 70, adenosyl homocysteine hydrolase and pectin esterase were expressed and identified in the AF during the interaction with *T. virens*. These proteins have been previously reported as part of the plant immune response pathways by activation of microbe elicitors (Kawalleck et al., 1992; Lionetti et al., 2007; Maimbo et al., 2007; Balmer et al., 2013); suggesting that maize roots are sensing *T. virens* elicitors, for example, chitin, and are responding to *T. virens* colonization.

The protein β-xylosidase (TV_29366) from *T. virens* was confirmed in this analysis, suggesting the secretion of hydrolytic enzymes into the apoplast by *T. virens*. The precursor of β-xylosidase has been reported as a virulence factor of *Sclerotinia sclerotium* (Yajima et al., 2009), and it has been observed previously in the secretome of *T. virens* interacting with maize roots under hydroponic conditions (Lamdan et al., 2015). β-xylosidase participates in the hydrolysis of xylan, one of the major polysaccharides present in plant cell walls.

### Identification of apoplastic proteins by gel-free proteomic technology

Using the gel-free shotgun proteomics approach, a total of 148 and 177 proteins were identified in un-inoculated and inoculated roots, respectively.

A number of cytosolic proteins were identified in this study which may suggest levels of contamination by cytoplasm from both organisms despite a low level of cytoplasm biomarker being detected (<1.5% of total MDH activity). Similar results were observed by Dragišić Maksimović et al. (2008), using the vacuum-infiltration technique to extract AF from maize roots. Other studies have identified classical cytoplasmic proteins as part of the secretome of different organisms, for example, plants and fungi (Agrawal et al., 2010; Kim et al., 2013). Techniques used for the isolation of APs may induce mechanical damage to host cells leading to cell breakdown and possible contamination by cytoplasmic proteins, which is a constraint in plant-microbe secretomics.

A comprehensive filter-pipeline was used in this study to identify and select putative secreted proteins from both organisms using available prediction software and literature. Nevertheless, proteins that were discarded as secreted proteins in this study may have also an important function during *T. virens-*maize interaction.

### Secretion systems of apoplastic proteins during *T. virens*-maize interaction

Identification of potential secreted-APs from maize under both conditions, suggests that maize roots secrete APs by using both conventional (proteins containing a signal peptide) and unconventional secretion systems which include the leaderless secretory proteins (LSPs). These findings have been previously reviewed in plant models by Yadav et al. (2015). Interestingly, *T. virens* seems to induce the secretion of LSPs; the plant apoplastic LSPs increased from 26% in the absence of the fungus to 55% when the fungus was present. These changes in the population of host secreted proteins during fungal colonization has been reviewed by Agrawal et al. (2010) in different plant models, suggesting that plant LSPs are involved in the defense/stress responses against microbial invasions. This may suggest that maize roots are responding to *T. virens* colonization by secreting proteins related to defense pathways through conventional and unconventional secretion systems.

Remarkably, 54% of the 92 identified *T. virens* secreted-APs lacked a signal peptide (Supplementary Figure 4B). In fungi under biotic conditions the secretome population includes LSPs and reveals several unknown mechanisms of secretion that are active during the microbe-plant interaction (Girard et al., 2013). It was expected that different mechanisms of secretion would be found in *T. virens* during the interaction. This secretion pattern has previously been reported during the detrimental interaction between *M. oryzea* and rice (Kim et al., 2013), where 48% of the total secretome identified corresponded to LSPs. Similar results were observed by Weber et al. (2012) during the secretome analysis of the fungus *Paracoccidioides*; where they predicted that 52% of identified secreted proteins are released by non-classical secretion systems. The exact mechanisms of secretion for leaderless secretion pathways remain largely unknown for plant-microbe interactions.

### Maize secretome: the influence of *T. virens* colonization

Functional analysis of the maize secretome present in the apoplast during colonization by *T. virens* showed that *T. virens* is inducing several changes in plant metabolism and activating pathways of stress responses against biotic factors. Interestingly, oxidoreduction activity seems to be reduced in the presence of *T. virens* in the apoplast (Figure 3B). Similar results were observed in the transcriptome analysis after 5 d interaction performed by Lawry (2016), where genes involved in plant defense responses were identified, implying that maize roots are responding to fungal colonization and vice versa.

#### Detoxifying and ROS related proteins influenced by *T. virens*

A larger number of proteins were observed during non-interaction conditions compared with inoculated roots, where a reduction in secreted proteins was observed in roots interacting with *T. virens*; for example, proteins belonging to the peroxidase family. Peroxidases are principal components in plant defense responses against pathogens either by cell wall reinforcement and/or for inducing plant oxidative bursts in the apoplast (Mehdy, 1994), creating unsuitable conditions for microbe interactions.

Differences were found in the secretion of peroxidases in this study; where 12 were secreted in the control roots; this number reduced to six during the *Trichoderma*-plant interaction, three of which were unique to the inoculated roots (A0A1D6N0K3_MAIZE; A0A1D6E530_MAIZE; K7VH58_MAIZE) (Table 1). This suggest that these latter peroxidases are part of the immune respond of maize to *T. virens* colonization. Remarkably, other peroxidases (B4FG39_MAIZE and B6TM55_MAIZE) were significantly reduced in their abundance when *T. virens* was present (Supplementary Figure 5). These results demonstrate that recognition of the endophyte *T. virens* by resistance proteins involves plant defense mechanisms and that this reduction in plant defense molecules such as peroxidases suggests that *T. virens* may be re-shaping plant secretome responses as a mechanism to suppress plant immunity. In the symbiotic interaction between *Glomus mossea* and tobacco roots oxidative reduction activity plays an important role, activity of ascorbate peroxidases was increased in mycorrhizal roots at earlier stages, but after the interaction is established the fungus is able to diminish plants immune responses (Blilou et al., 2000).

Additionally, we identified proteins in un-inoculated maize such as superoxide dismutase [Cu-Zn] (SODC5_MAIZE), peroxiredoxin-2B (B4FN24_MAIZE), thioredoxin (Trxh1) (B6SHW5_MAIZE), and glutathione S-transferase (B4FSR6_MAIZE; GSTF4_MAIZE; B6TL20_MAIZE) that are involved in detoxification of the apoplast reducing ROS, but in inoculated roots the peroxiredoxin, thioredoxin and one glutathione S-transferase weren't present. ROS play a major role in plant defense. Therefore, to maintain a balance during the plant-microbe interaction, all these enzymes are considered necessary to confer resistance to oxidative stresses. Interestingly, antioxidant activity is reduced in maize roots during the interaction with *T. virens*, suggesting that alterations in redox activity are influenced by fungal colonization.

#### Pathogenesis related proteins secreted in presence of *T. virens*

Plant defense-related proteins were also identified in the secretome for both conditions. Pathogenesis related proteins (PRs) are produced in plants in the event of microbial recognition, and involve antimicrobial activity. Most PRs are induced through jasmonic acid (JA), salicylic acid (SA) and ethylene (ET) defense signaling (van Loon et al., 2006). Extracellular defense-related proteins are considered the first line of defense against invading attackers before tissue penetration takes place. Several PR-like proteins were identified in the apoplast from un-inoculated and inoculated plants, for example, chitinase, endochitinase B, nine peroxidases (PR-9), two ribonucleases (PR-10), two proteinase inhibitors (PR-6), and an osmotin-like protein (PR-5) (Table 1 and Supplementary Table 2). These proteins may be considered as part of basal defense mechanisms in maize, because they were present in both conditions. Additionally, other defense-related proteins were identified that were probably induced by the mechanical wounding provoked by the manipulation of the samples, for example, the Bowman-Birk type wound-induced proteinase inhibitor. Hence we cannot discard that some of previous proteins described were activated through this process, given that several-defense proteins are induced during wounding or cold stress (van Loon et al., 2006).

In the presence of *T. virens*, maize roots expressed an increase of different PR-like proteins. APs such as Barwin superfamily protein (B6SH12_MAIZE), cysteine-type endopeptidase (A0A1D6ICV7_MAIZE), PR-10 (A0A1D6JZU3_MAIZE), three peroxidases (A0A1D6N0K3_MAIZE; A0A1D6E530_MAIZE; K7VH58_MAIZE), proteinase inhibitor (Pis7) (Q42420_MAIZE), serine-type endopeptidase inhibitor (K7U234_MAIZE), and osmotin-like protein (A0A096PW84_MAIZE) were activated during fungal colonization. This suggests that these proteins are specifically activated during *T. virens* recognition. Comparable findings during the three-way interaction between *Trichoderma atroviride*, a host plant and a fungal pathogen were identified, where PRs were up-regulated when *T. atroviride* was interacting with the plant (Marra et al., 2006). Although maize responded by constitutive expression of various PRs, they did not affect colonization by the beneficial *T. virens*. Similar findings were observed during the beneficial relationship between the mycorrhizal fungus *G. mosseae* and its host plant (Vierheilig et al., 1995). Remarkably, *T. virens* may directly affect the secretion of specific PRs such as ribonuclease (B4FBD6_MAIZE) and PR-10 (Q29SB6_MAIZE) as their abundance in the apoplast was reduced; however, the plant may counter attack by activating other PR proteins, for example, serine-type endopeptidase inhibitor (K7U234_MAIZE) to target proteins secreted by *T. virens* in the apoplast (Supplementary Figure 5). The serine-type endopeptidase inhibitor was identified as the only protein whose abundance was higher in presence of *T. virens* (Supplementary Figure 5).

#### Secreted proteins involved in hormone signaling cell wall modification, nutrient acquisition, protein modification and metabolism

Proteins involved in phytohormones signaling were identified. These were predominantly from the ethylene pathway, including proteins responsible for the biosynthesis of precursor components such as methionine synthase (COP5Y3_MAIZE), adenosylhomocysteinase (COPHR4_MAIZE) and the enzyme 1-aminocyclopropane-1-1-carboxylate (ACC) oxidase (COPEP2_MAIZE) which catalyzes the final stage of ethylene biosynthesis. Levels of adenosylhomocysteinase were influenced by the presence of *T. virens* and ACC oxidase was only expressed in inoculated plants suggesting that one strategy of the fungus is the manipulation of plant immunity responses via the ethylene pathway. In the pathosystem *Arabidopsis-Pseudomonas syringae*, Kaffarnik et al. (2009) observed that the protein abundance of methionine synthase was dramatically influenced in the apoplastic region and not intracellularly by the bacterial effector-like proteins.

Other proteins with relevance in the AF that were identified are involved in cell wall modification and nutrient acquisition such as glycosyl hydrolases (GHs) proteins, signal transduction and secondary messengers, such as, 14-3-3 protein and calmodulin, respectively (Cui et al., 2005; Lozano-Durán and Robatzek, 2015); proteins that participate in protein modification such as heat shock 70 protein (chaperone) and protein disulphide-isomerase (Pdil 1) involved in correct protein folding essential for protein functionality (Park and Seo, 2015; Porter et al., 2015); and ribosomal proteins, cyclophilin and proteins involved in lipid transport and secondary metabolism that have potential roles in defense/stress responses (Agrawal et al., 2010) were also observed in the AF. Under unstressed conditions these APs are involved in cell wall modification or maintenance (Albenne et al., 2013), whereas upon pathogenic attack they are specifically involved in cell wall remodeling and reinforcement (Hamann, 2012).

Overall, all the identified proteins above play important roles in basal and induced defense/stress responses, metabolism, signaling and protein modifications in plants (Agrawal et al., 2010) and have been previously reported as part of the apoplastic secretome *in-planta* (Gupta et al., 2015), where several plant response pathway were activated or inactivated by the presence of *T. virens*.

### *T. virens* secretome during the interaction with maize roots

Diverse mechanisms are involved in the *Trichoderma-*plant interaction during the process of root colonization (Harman, 2006). In this study several protein families were identified that may have a direct influence on *T. virens* colonization and plant defense manipulation, which correlates with the findings previously reported by Lamdan et al. (2015), where protein families such as glycosyl hydrolases, antioxidant proteins, small secreted cysteine rich proteins and secondary metabolism proteins were secreted in the presence of maize roots.

#### Glycosyl hydrolase (GH) protein family (cell wall modification)

Secretion of both endophyte and host glycosyl hydrolases have an important role either promoting successful colonization via degradation of the host cell wall, or resisting microbe invasion through the reinforcement of host defenses via cell wall maintenance during early stages of the interaction. Seven GH proteins were identified including two -galactosidases (TV_42143 and TV_78372) that hydrolyse galactose-rich polysaccharides in plant cell walls (Ranwala et al., 1992), galactose mutarotase-like protein (TV_87809) that participates during galactose metabolism, -glycosidase (TV_71600) involved in catabolism and turnover of plant N-glycans (Minic, 2008), -glucosidase (TV_110754) involved in cellulose degradation (Tiwari et al., 2013), -xylosidase (TV_29366), and cellobiohydrolase (TV_90504) for degradation of xylan and cellulose, respectively (Nummi et al., 1983; Biely, 1985). Interestingly, the secretion of the glycosyl hydrolases and cell wall-degrading enzymes (CWDEs) identified in this study are mediated through the Golgi-ER secretion pathway. In addition, all identified enzymes were transcriptionally up-regulated after 5 d interaction with maize roots compared with axenic conditions (Lawry, 2016). CWDEs have also been proposed to act as virulence factors (effectors) in *U. maydis* and *M. oryzea*; however, the exact role they play in plant immunity is not known (Kubicek et al., 2014). The results suggest when *T. virens* encounters its host plant root system, to overcome the barrier of the plant cell wall and successfully penetrate and colonize internally, *T. virens* may secrete enzymes into the apoplast such as glycosyl hydrolases and CWDEs which focus on the degradation of cell wall polymers necessary for colonizing host tissues.

#### Antioxidant secreted proteins

The oxidative burst is one of the most critical events upon plant recognition of plant pathogens (Heller and Tudzynski, 2011). This reaction is activated by a rapid production of ROS in the apoplast such as superoxide, hydroxyl radical and hydrogen peroxide which are also involved in plant response signaling. In addition, ROS activate physiological changes in the plant such as cell wall strengthening. Although ROS induction is transient *in-planta*, the compounds are highly reactive and can cause detrimental oxidation of essential macromolecules causing host cell damage and triggering hypersensitive response (HR), if they are not controlled (Nanda et al., 2010). ROS are not only toxic, but are also signaling molecules involved in growth and environmental adaptation (Marschall and Tudzynski, 2016). Antioxidants can protect the cell from oxidative damage by scavenging the ROS. Therefore, cellular redox homeostasis is important to maintain plant-microbe symbiotic interactions (Marschall and Tudzynski, 2016). In this study, *T. virens* secreted proteins were identified that work as detoxification enzymes. The enzyme superoxide dismutase [Cu/Zn] (TV_183329) was present in the apoplast and is involved in the breakdown of ROS molecules protecting fungal integrity. Superoxide dismutase is an essential molecule during plant microbe interactions. During the beneficial relationship between the fungal endosymbiont *Neotypodium lolii* and ryegrass, superoxide dismutase is necessary for limited host defense and normal endophytic growth (Zhang et al., 2011). The protein catalase-peroxidase haem (TV_215514) was also identified. This enzyme exhibits both catalase and peroxidase activity, and provides protection against oxidative stress dismutating H_2_O_2_ to O_2_ + H_2_O. In the fungus *M. oryzea*, the secreted catalase-peroxidase (CPXB) confers resistance to H_2_O_2_ accumulated in epidermal cells of rice, but is not essential for pathogenicity (Tanabe et al., 2011). As part of the antioxidant protein arsenal, the enzyme glutathione reductase was found, which is required for protection against oxidative stress. Glutathione reductase (TV_ 54541) plays an essential role in rice blast disease, facilitating biotrophic colonization of host cells and by suppression of host ROS accumulation (Fernandez and Wilson, 2014). In the *Gluconacetobacter diazotrophicus*-rice system glutathione reductase was determined to be crucial for endophytic colonization (Alquéres et al., 2013). Also identified was the protein pyridine nucleotide-disulphide oxidoreductase (TV_138628) which is associated with antioxidant activity. All these antioxidant proteins secreted by *T. virens* have been related to inactivation of ROS during plant-microbe interactions. Moreover, the GPI-anchored protein Ecm33-like (TV_72615) from *T*. virens was identified in the secretome. Ecm33 protein being reported as a GPI-anchored protein that attaches into the plasma membrane in *Beauveria bassiana* and *Metarhizium robertsii*, where it contributes to multi-stress tolerance against oxidant molecules, fungicide and osmotic stress (Chen et al., 2014). In *T. virens*, Ecm3 was found by Lamdan et al. (2015) as part of the secretome in the presence of maize roots. This may suggest that during colonization Ecm33-like protein may play an important role due to its properties as a multi-stress tolerance molecule, protecting *T. virens* hyphae against the oxidative burst created by host cells. Overall, the results presented here suggest that antioxidant secreted proteins collaborate in the suppression of basal resistance through the regulation of the cellular redox state, and by modifying gene expression in the host plant to maintain symbiosis between *T. virens* and maize.

#### Secreted proteins involved in secondary metabolism biosynthesis

Fungal secondary metabolites (SMs) are classified into four classes: polyketides, terpenoids, shikimic acid derived compounds, and non-ribosomal peptides. During plant-microbe interactions, secondary metabolites such as phytohormones and toxins play major roles in the regulation of plant metabolic and defense response processes (Pusztahelyi et al., 2015). In addition, fungal secondary metabolites can shape fungal-plant interactions in a similar way as effector proteins (Pusztahelyi et al., 2015). The protein (S-adenosylmethionine (SAM) synthase (TV_87758), involved in the biosynthesis of the precursor of ethylene (ET) was identified and, ET is one of the phytohormones involved in plant defense responses (Broekgaarden et al., 2015). Interestingly, SAM synthase is involved in the ET biosynthesis pathway and is predicted to be secreted into the apoplast through a non-classical secretion system, where has been reported to be secreted by the presence of host roots (Lamdan et al., 2015). Ethylene is a crucial phytohormone for plant defense responses during the plant-microbe interactions, where successful plant symbionts have evolved different strategies to manipulate plant responses by affecting the ethylene pathway. For example, the small secreted protein SP7 from the arbuscular mycorrhizal *Glomus intraradices* is translocated into the cell nucleus and interacts with host ethylene-responsive transcription factor (ERF19) interfering with the defense cascade, thereby promoting mycorrhizal colonization (Kloppholz et al., 2011). In *T. harzianum*, activation of the enzyme ACC deaminase promotes root elongation of canola seedlings by modulating ET levels in the host plant (Viterbo et al., 2010). Results suggest that *T. virens* may influence ET levels in the apoplast allowing *T*. *virens* to colonize the intercellular spaces without triggering a major immune response.

Additionally, the proteins S-adenosyl-L-methionine methyltransferase (GliN) (TV_91355) and aminotransferase (GliI) TV_53497 which are involved in gliotoxin biosynthesis and belong to the gliotoxin cluster in *T. virens* (Mukherjee et al., 2012) were identified. Gliotoxin is a fungistatic mycotoxin that confers protection to oxidative stress and participates in the mycoparasitism but does not have an influence in *T. virens* root colonization (Vargas et al., 2014). The role of mycotoxins in plant microbe interactions are mainly described as virulence or pathogenicity factors. It will be necessary to elucidate the function of *Trichoderma* toxins during host colonization, which may act as neutralizers of plant defense mechanisms (Pedras and Ahiahonu, 2005).

#### Putative effector-like proteins

To establish a beneficial or detrimental interaction plant microbes deliver effector-like proteins into host tissues, where they play a central role in the suppression of plant defense responses and modulate plant physiology by reprogramming metabolic processes of colonized tissues (Doehlemann et al., 2014; Mendoza-Mendoza et al., 2018). Effector-like proteins are defined as stable small secreted proteins normally ≤ 300 amino acids as although larger proteins have been identified that play similar roles (Lo Presti et al., 2015). Effector-like proteins are secreted either into the apoplast space or translocated into the plant cells (Lo Presti et al., 2015). Putative effector-like proteins were identified in the secretome of *T. virens* during the interaction with maize roots. The effector-like proteins were divided into subgroups according to their predicted functions.

##### Thioredoxin-like proteins

As mentioned above, the oxidative burst and the progressive induction of ROS have a major impact during plant microbe interactions; hence their homoeostasis in the apoplastic region is necessary for a long-term relationship. Two thioredoxins (TV_81963 and TV_216458) and one disulphide-isomerase (TV_11061) were identified which participate in the rearrangement of -S-S- bonds in proteins. These enzymes are secreted into the apoplast by *T. virens* upon colonization of maize roots and may function as putative effector-like proteins, reducing ROS activity in the apoplast and, in parallel, diminishing the plant defense reactions.

Several studies have summarized the importance of thioredoxins in plant-microbe interactions. The thioredoxin GBNRx1 from *V. dahlia* plays a crucial role in the apoplastic immune response functioning in apoplastic ROS scavenging in cotton (Li et al., 2016). The thioredoxin system has a major influence in *Botrytis cinerea* pathogenicity protecting it from oxidative stress (Viefhues et al., 2014). The thioredoxin complex influences several cellular processes by affecting protein folding or their activity by thiol-disulphide redox control (Arnér and Holmgren, 2000). In *Ralstonia solani*, the thioredoxin TRX2 is necessary for the activation of the effector RipAy that degrades glutathione which is involved in the plant immune response (Mukaihara et al., 2016). Thioredoxins are also involved in the bacterial-plant interactions where their plays a role in melanin synthesis and contributes to signal transduction during symbiotic nitrogen fixation, giving resistance to oxidative stress (Castro-Sowinski et al., 2007). Results suggest that *T. virens* may use antioxidant-secreted proteins that can act as effector-like proteins to control ROS levels in the apoplast which diminishes the plant immune response thus enabling fungal colonization.

##### Small secreted cysteine proteins (SSCPs)

Small secreted cysteine proteins (SSCPs) are widely distributed in plant symbionts. *Trichoderma* spp. (*T. virens, T. atroviride*, and *T. reesei*) contain around 173 SSCPs. Of these, 129 are specific to *Trichoderma* species and around 25 are unique having no BLAST matches (Lawry, 2016). Three SSCPs were identified, including Sm1 (TV_110852), SSCP1 (TV_92810), and SSCP2 (TV_111995) in the secretome from *T. virens* in the apoplast. The small protein (Sm1) belongs to the cerato-platanin family and was reported as an elicitor that induces systemic disease resistance through activating the JA pathway in the host plant (Djonović et al., 2007). However, the specific role of Sm1 during *T. virens* colonization is not known. Several paralogs of Sm1 have been identified: Sm2, Sm3, and Sm4 (Crutcher et al., 2015). Sm2 is highly expressed in the presence of maize roots compared with the other homologs, is involved in colonization and is more important than Sm1 in the promotion of plant protection (Gaderer et al., 2015), however we did not identify Sm2 in the *T. virens* secretome. The protein SSCP1 exhibits an 8 cysteine-containing CFEM domain was reported in the secretome of *T. virens* in the presence of maize roots (Lamdan et al., 2015). SSCP1 was predicted to act as a negative effector, directly affecting the defense level in the plant. The SSCPs in *T. virens* may play essential roles additional to elicitor and effector functions such as cell surface receptors, signal transducers, or adhesion molecules during colonization. Few of the SSCPs have been characterized so far in *Trichoderma* (Djonović et al., 2007; Crutcher et al., 2015; Gaderer et al., 2015; Lamdan et al., 2015). Further research is required to understand the redundancy and abundance of SSCPs in *T. virens* during the symbiotic relationship with it host plant.

##### Protein inhibitors (PIs)

The proteinase inhibitor I9 (TV_216375) was identified in the *T. virens* secretome. This protein belongs to the protease inhibitor superfamily, which are responsible for the modulation of folding and activity of the peptidase pro-enzyme. We hypothesize that PIs may be indispensable for the inactivation of plant defense secreted proteins in the apoplast, enabling fungal colonization and inactivation of plant defenses. Several apoplastic effectors in fungal plant pathogens with protease activity have been described during successful infection, for examples, the effector Pit2 in *U. maydis* functions as an inhibitor of maize cysteine proteases, and is required for fungal virulence and suppression of host immunity (Mueller et al., 2013). Other examples are the protease inhibitor Avr2 secreted by the fungal plant pathogen *C. fulvum* that targets the tomato defense protease Rcr3 (Song et al., 2009), and recently, a candidate effector had been predicted in *S. sclerotorium* that has a conserved serine protease I9 domain, which may be necessary to suppress host resistance (Guyon et al., 2014). In the *T. virens*-maize interaction different proteases were observed such as aspartic-type endopeptidase (A0A1D6F8J3_MAIZE) and a cysteine-type proteinase (A0A1D6ICV7_MAIZE) secreted by maize that were expressed during colonization. For that reason, PIs may be essential to inactivate or block plant immune proteases.

#### Peroxidase levels influenced by *T. virens*

Peroxidases are defense-related enzymes that are induced in the presence of plant associated microbes triggered by elicitor-induced signal transduction pathways (Almagro et al., 2009). The fact that peroxidase activity levels were significantly reduced in the presence of *T. virens* at 5 d interaction (Figure 5A) may suggest that fungal colonization does not activate stronger immune responses in maize roots at this development stage of the interaction. Moreover, reduction in peroxidase activity may directly influence lignification and suberization of cell walls and crosslinking of cell wall structural proteins allowing internal colonization by *T. virens* as shown in Figure 2D, due to their role in different physiological process and defense reactions such as cell wall reinforcement (Hiraga et al., 2001; Almagro et al., 2009). In poplar plants, peroxidase activity was influenced by the ectomycorrhizal *Paxillus involutus* dependent on the compatibility of the isolate, showing that during compatible interactions changes in the level of peroxidases were not observed, but under incompatible interaction the levels were significantly higher compared to the control plants (Gafur et al., 2007).

ROS scavenging enzymes secreted by *T. virens* into the apoplast, such as glutathione reductase (TV_54541), catalase-peroxidase haem (TV_215514), superoxide dismutase [Cu-Zn], thioredoxin reductase (TV_81963) and thioredoxin-related protein (TV_216458) help overcome the oxidative burst, which may have an effect in the activation of robust plant defense reactions including plant peroxidases. It has been demonstrated that production of fungal ROS can result in the suppression of endophyte growth in the host plant, which is critical in the mutualistic *Epichloë festuce* and grass interaction (Tanaka et al., 2006). Therefore, we suggest that *T. virens* secretes detoxifying enzymes into the apoplastic as a strategy to maintain ROS stability to mitigate the impact of stress and to manipulate further plant immune responses diminishing the effect of peroxidases in the apoplast.

## Conclusions

**Figure 6:**
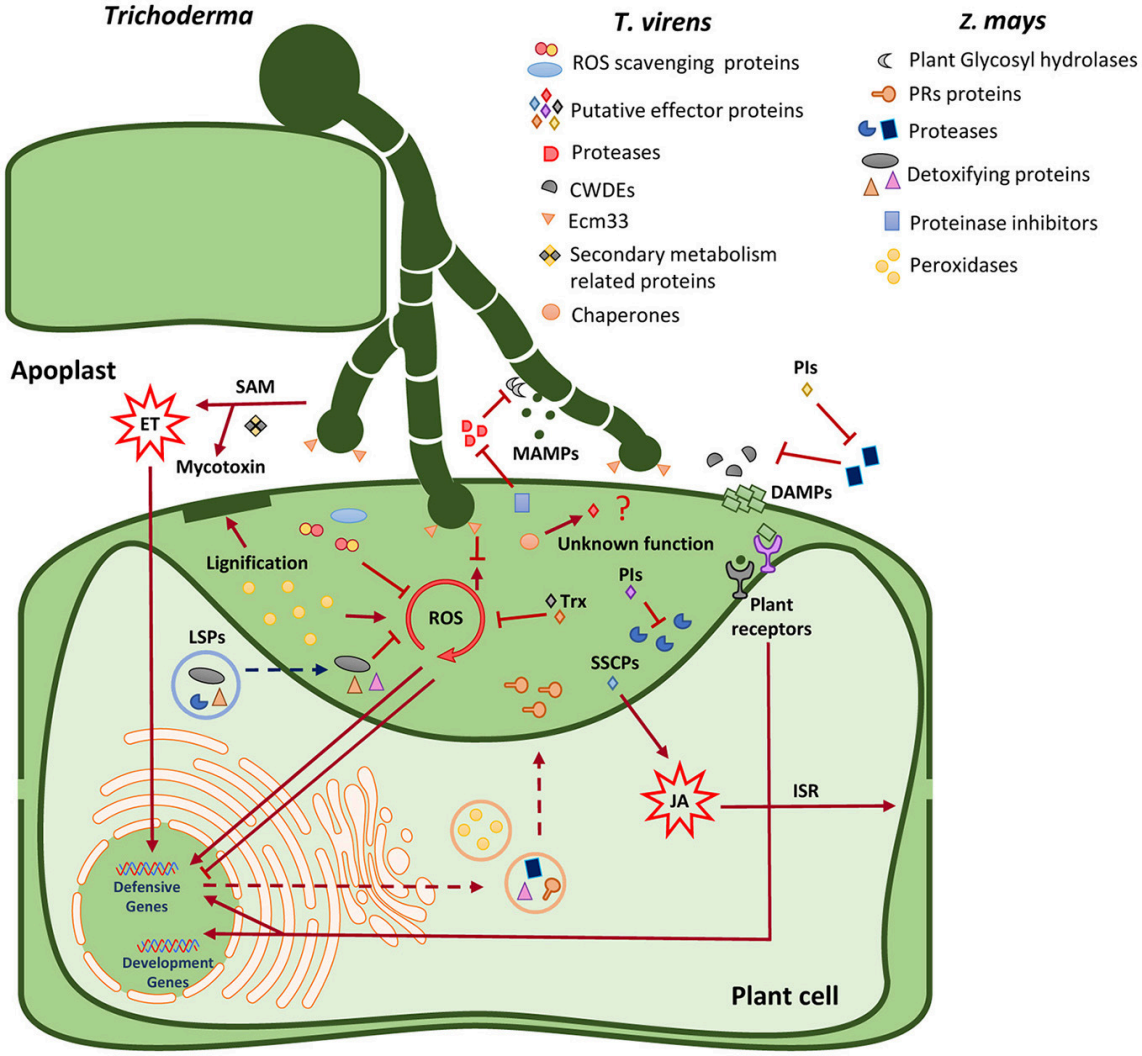
Model of apoplastic proteins identified in the *T. virens-*maize interaction. Location and putative function of apoplastic proteins secreted by *T. virens* and maize. ET, ethylene; JA, jasmonic acid; ISR, induced systemic resistance; ROS, reactive oxygen species; MAMPs, microbial-associated molecular patterns; DAMPS, damage-associated molecular patterns; CWDEs, Cell wall degradative enzymes; PRs, pathogenesis-related proteins; PIs, proteinase inhibitors; Trx, thioredoxin; LSPs, leaderless secretory proteins; SAM, S-adenosyl-l-methionine; SSCPs, small secreted cysteine-rich proteins.

In closing, this study reveals possible mechanisms necessary for *T. virens* endophytism (Figure 6). We identified that host cell wall degradation and modification are essential during internal colonization. Several pathways are activated during the interaction. In particular, redox homeostasis is crucial to maintain a controlled environment in the apoplast. We propose that both organisms secrete proteins into the apoplast via conventional and unconventional secretory mechanisms. We identified putative effector-like proteins secreted by *T. virens* that may lead the symbiotic relationship by the suppression of plant immune pathways, for example, peroxidase activity. This study lays the foundation for future studies for a better understanding of plant-fungus interactions, particularly at the proteomic level.

## Author contributions

GN-L and MM performed experimental work; GN-L, AM-M, JS, CW, and DG designed the experiments; GN-L, AM-M, JS, CW, DG, CE, and MM discussed and interpreted the results; GN-L, AM-M, JS, and CW designed the research; AM-M and DG contributed to chemicals and scientific advice; GN-L, AM-M, and JS wrote the paper. All authors reviewed the final version of the paper.

### Conflict of interest statement

The authors declare that the research was conducted in the absence of any commercial or financial relationships that could be construed as a potential conflict of interest.
