# Supplementary material for: The Apoplastic Secretome of Trichoderma virens During Interaction With Maize Roots Shows an Inhibition of Plant Defence and Scavenging Oxidative Stress Secreted Proteins
Source: Front Plant Sci. 2018 Apr 5;9:409. doi: 10.3389/fpls.2018.00409 (PMC5896443; doi:10.3389/fpls.2018.00409)
Supplement: Supplementary file 3 [file Presentation1.pdf]

Supplementary methods

1.1.1 Sorption method

Fungal material was removed from the root surface as mentioned above. The plants were placed immediately on germination paper (30 cm x 45 cm; Anchor Paper Company, USA) soaked with sterile Hoagland´s solution. For collection of root AF, filter paper strips (10 mm x 5 mm; GE Healthcare, IL, USA) were used as sorption media. The strips were washed in methanol and bi-distilled water and dried before being applied to the root surface (Dragisic Maksimovic et al., 2014). Two filters per plant were positioned onto the first 2 cm root surface and kept in place for 1 h. To prevent root drying, the remaining root system was covered with filter paper moistened with Hoagland´s solution. After 1 h, forty filter paper strips (equivalent to 20 plants) with absorbed AF were removed and placed into a microcentrifuge tube containing 1 mL SPB buffer solution supplemented with 0.3% (v/v) Pefabloc and 10 mM EDTA. Apoplastic proteins (APs) were extracted from the filter paper strips by vigorously vortexing the tubes for 5 min. The filters were then transferred to a new microcentrifuge tube. Both tubes, one containing filters and the other containing the buffer were immediately snap frozen in liquid nitrogen and stored at -80°C.

Supplementary Figures

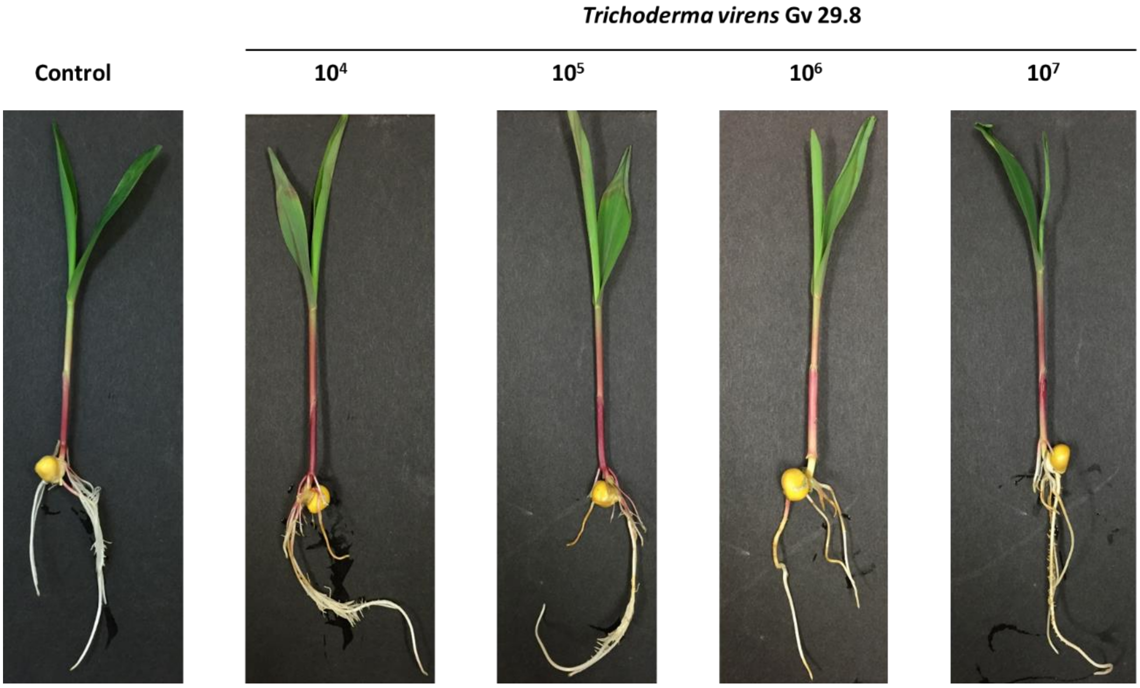

Supplementary Figure 1. Observations of phenotypic changes in the maize root system inoculated with different concentrations of *T. virens*. Maize roots showed brownish pigmentation on the root surface independently to different concentrations of inoculum (*T. virens*), starting from 10^4 to 10^7 spores, after 7 days interaction.

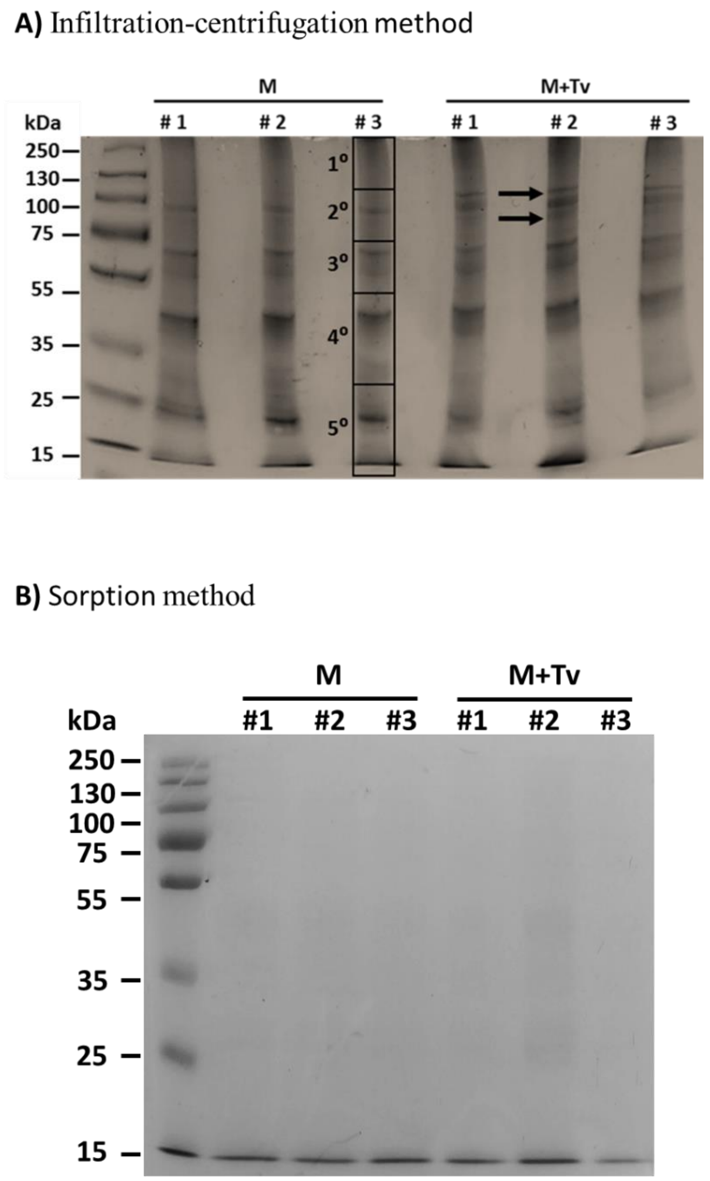

Supplementary Figure 2. Electrophoretic profile (1D-SDS-PAGE) of protein extracts of apoplastic fluid from maize primary roots. Three biological replicates of apoplastic proteins fractions from maize roots un-inoculated (M) and inoculated (M+Tv) with *T. virens* were separated by 1D-SDS-PAGE. **(A)** Apoplastic proteins isolated by infiltration-centrifugation method. Black squares represent the five sections that protein fractions were divided into MS identification; section 1 represents (250-120 kDa), section 2 (120-75 kDa), section 3 (75-50 kDa), section 4 (50-30 kDa), and section 5 (30-15 kDa). Black arrows indicate visible differences in protein fractions located in section 2 in inoculated compared with un-inoculated protein profiles**. (B)** Apoplastic proteins isolated by the sorption method. No visible protein fractions were observed in the samples.

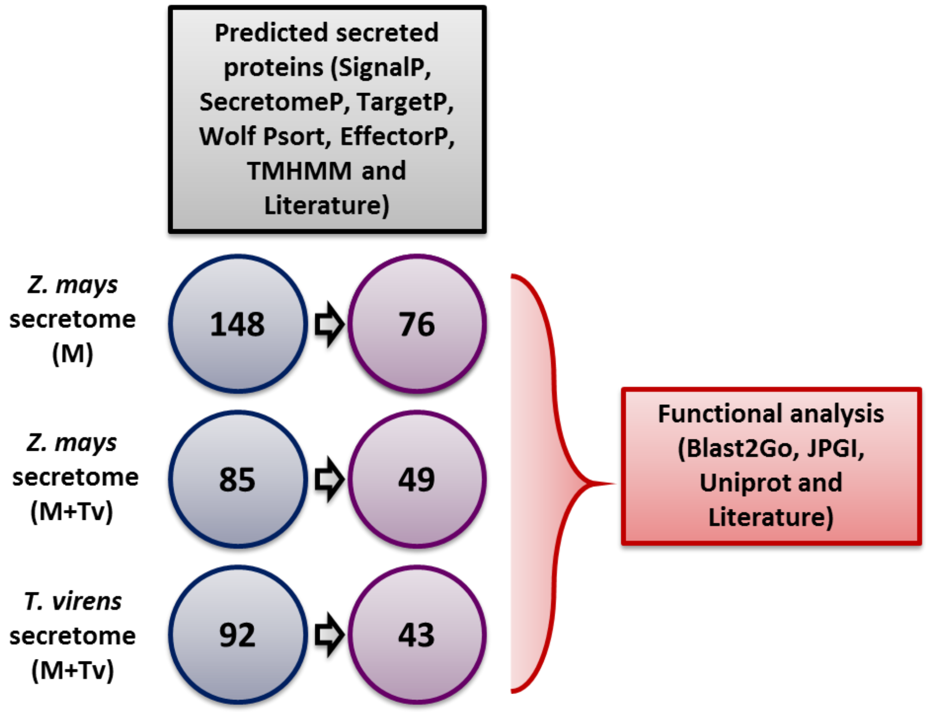

Supplementary Figure 3. Diagrammatic outline of the process used to detect and analyse putative secreted proteins by *T. virens* and maize during 5 days interaction. The secretome was obtained after isolation of apoplastic proteins during the *T. virens*-maize interaction. The secretome was examined with different prediction software to detect their secretion status (SignalP, SecretomeP, TargetP, Wolf Psort, EffectorP, TMHMM and literature). The predicted secreted proteins were then analysed for likely function based on their ontology, homology, and domains (Blas2GO, JPGI, Uniprot and literature).

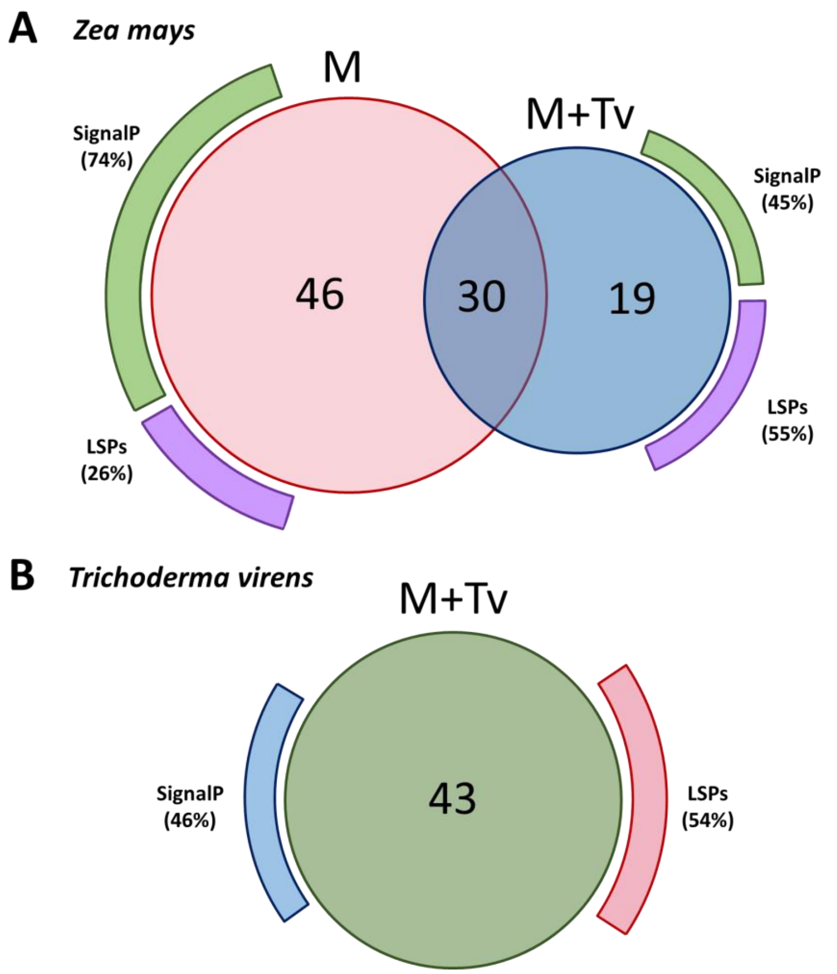

Supplementary Figure 4. **Apoplastic proteins secreted by *T. virens* and maize.** Delineation of apoplastic proteins that are secreted by classical and non-classical secretion systems. **(A)** Relationship of apoplastic proteins identified from maize in un-inoculated (M) and inoculated (M+Tv) roots and their secretion system prediction. **(B)** Apoplastic proteins identified from *T. virens* in inoculated roots and their secretion system prediction. Abbreviation: leaderless secretion proteins (LSPs).

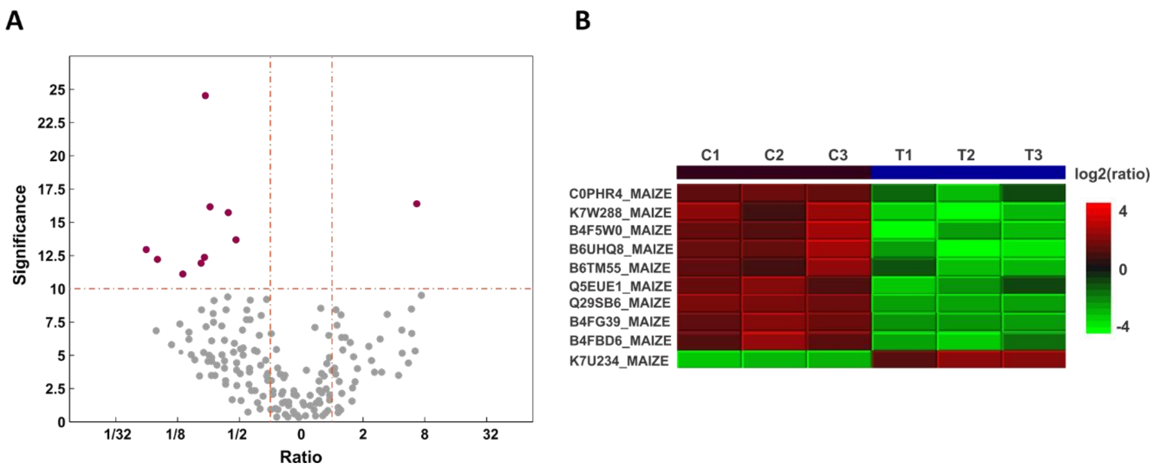

Supplementary Figure 5. Label-free quantification of apoplastic proteins during the *T. virens*-maize interaction at 5 days. (A) Volcano plot for maize proteins statistically significant (significance ≥ 10; fold change ≥ 1.5) during interaction. (**B)** Protein profile heat map of the most significantly different maize proteins comparing un-inoculated (C) and inoculated (T) plants. Cell colour represents the log2 (ratio) to the average are across different samples (C1, C2, C3, T1, T2 and T3).
